# Crystal Structure of a Yeast Aquaporin at 1.15 Å Reveals a Novel Gating Mechanism

**DOI:** 10.1371/journal.pbio.1000130

**Published:** 2009-06-16

**Authors:** Gerhard Fischer, Urszula Kosinska-Eriksson, Camilo Aponte-Santamaría, Madelene Palmgren, Cecilia Geijer, Kristina Hedfalk, Stefan Hohmann, Bert L. de Groot, Richard Neutze, Karin Lindkvist-Petersson

**Affiliations:** 1 Department of Chemistry, Biochemistry and Biophysics, University of Gothenburg, Göteborg, Sweden; 2 Computational Biomolecular Dynamics Group, Max Planck Institute for Biophysical Chemistry, Göttingen, Germany; 3 Department of Cell and Molecular Biology, University of Gothenburg, Göteborg, Sweden; Brandeis University, United States of America

## Abstract

Aquaporins are transmembrane proteins that facilitate the flow of water through cellular membranes. An unusual characteristic of yeast aquaporins is that they frequently contain an extended N terminus of unknown function. Here we present the X-ray structure of the yeast aquaporin Aqy1 from *Pichia pastoris* at 1.15 Å resolution. Our crystal structure reveals that the water channel is closed by the N terminus, which arranges as a tightly wound helical bundle, with Tyr31 forming H-bond interactions to a water molecule within the pore and thereby occluding the channel entrance. Nevertheless, functional assays show that Aqy1 has appreciable water transport activity that aids survival during rapid freezing of *P. pastoris*. These findings establish that Aqy1 is a gated water channel. Mutational studies in combination with molecular dynamics simulations imply that gating may be regulated by a combination of phosphorylation and mechanosensitivity.

## Abstract 2

Atomic-resolution X-ray crystallography, functional analyses, and molecular dynamics simulations suggest a novel mechanism for the regulation of water flux through the yeast Aqy1 water channel.

## Author Summary

All living organisms must regulate precisely the flow of water into and out of cells in order to maintain cell shape and integrity. Proteins of one family, the aquaporins, are found in virtually every living organism and play a major role in maintaining water homeostasis by acting as regulated water channels. Here we describe the first crystal structure of a yeast aquaporin, Aqy1, at 1.15 Å resolution, which represents the highest resolution structural data obtained to date for a membrane protein. Using this structural information, we address an outstanding biological question surrounding yeast aquaporins: what is the functional role of the amino-terminal extension that is characteristic of yeast aquaporins? Our structural data show that the amino terminus of Aqy1 fulfills a novel gate-like function by folding to form a cytoplasmic helical bundle with a tyrosine residue entering the water channel and occluding the cytoplasmic entrance. Molecular dynamics simulations and functional studies in combination with site-directed mutagenesis suggest that water flow is regulated through a combination of mechanosensitive gating and post-translational modifications such as phosphorylation. Our study therefore provides insight into a unique mechanism for the regulation of water flux in yeast.

## Introduction

Transmembrane water flux is fundamental to the physiology of all living organisms, yet biological membranes display only limited intrinsic water permeability. Cells thus control and maintain water homeostasis using aquaporins, which facilitate the selective movement of water (orthodox aquaporins) or other small molecules such as glycerol or urea (aquaglyceroporins) [1]. These solutes pass through aquaporins at almost the diffusion rate yet the pore simultaneously prevents the leakage of protons [2]–[4]. Human and plant aquaporins are frequently regulated post-translationally by phosphorylation [5] or by biophysical stimuli such as changes in pH [6],[7].

Unicellular organisms, which have large surface-to-volume ratios, tightly regulate the flow of water and osmolytes during different stages of growth and under conditions of stress [8]. Yeast aquaporins are suggested to be regulated at the transcription level [9], and in addition a yeast aquaglyceroporin has been reported to be post-translationally regulated by phosphorylation [10] or osmolarity [11]. In the well studied yeast *Saccharomyces cerevisiae*, the presence of aquaporins enhance the host's tolerance to rapid freezing [12] and aquaglyceroporins control the cellular osmolyte content following osmotic shock [13]. The physiological relevance of rapid freeze tolerance is explained by the formation of less intracellular ice crystals. During the freezing of cells in an aqueous medium, extracellular water will freeze faster than the intracellular water because of the higher osmolarity of the cellular content. Aquaporins facilitate the rapid out flow of water out of the cell and hence less intracellular ice forms within the organism, which results in less cellular damage [8].

*Pichia pastoris* is a methylotrophic yeast that was first isolated from chestnut trees [14] but is now widely used as a protein overproduction host [15]. Its genome encodes a single aquaporin, Aqy1, and no aquaglyceroporin. In comparison, the genome of *S. cerevisiae* encodes two aquaporins and two aquaglyceroporins. It is common among yeast and filamentous fungi to only have one orthodox aquaporin, but it is rather uncommon to lack aquaglyceroporins [9]. Nevertheless, the human fungal pathogen *Candida albicans*, which is carried by 80% of the world's population with no harmful effects but can be fatal in immunocompromised patients [16], also contains only a single aquaporin, which has high sequence similarity (67%) to Aqy1 of *P. pastoris*. Another striking feature of Aqy1 is that it contains an extended N terminus of unknown function, a characteristic that is frequently encountered among yeast aquaporins and aquaglyceroporins. Specifically, when compared with its closest human homologue aquaporin 1 (hAQP1), Aqy1 has 34 additional N-terminal residues.

There are currently nine reported high-resolution aqua(glycero)porin structures (<3.0 Å) within the protein structure database: three from bacteria [17]–[19], two from bovine [4],[20], one from human [21], one from sheep [22], one from spinach [23], and one from *Plasmodium falciparum* [24]. All the reported structures share a conserved overall fold with six transmembrane helices and five loops, where loops B and E dip into the channel and together form the seventh pseudo-helix. However, there are no reported structures of aquaporins from yeast or filamentous fungi. To better understand unique structural and functional characteristics of yeast aquaporins, we determined the structure of Aqy1 from *P. pastoris* at 1.15 Å resolution, which is the highest resolution structure reported to date for a membrane protein. At this resolution the anisotropic motions of water molecules and side-chains could be observed within the channel. Furthermore, the structure clearly establishes that the channel is gated, where a unique conformation of the N terminus is observed to cap the water channel entrance and thereby close the channel. This closure mechanism is distinct from that of a gated plant aquaporin, for which an extended D-loop caps the channel from the cytoplasm [23].

Measurements of the water transport activity using both proteoliposomes and spheroplasts of *P. pastoris*, showed that full length Aqy1 is active, whereas the N-terminal truncated form of Aqy1 has dramatically increased water conductance. From a combination of site-directed mutagenesis data and molecular dynamics simulations a unified picture emerges for the regulation of Aqy1, whereby the water conductance is suggested to be controlled by a combination of mechanosensitive gating and post-translational regulation by phosphorylation. Thus our findings for Aqy1 combine to establish that this orthodox aquaporin is gated by a unique mechanism intimately associated with the characteristic N terminus extension of the yeast aquaporins.

## Results

### Exceptionally High-Resolution Structure

**Figure 1:**
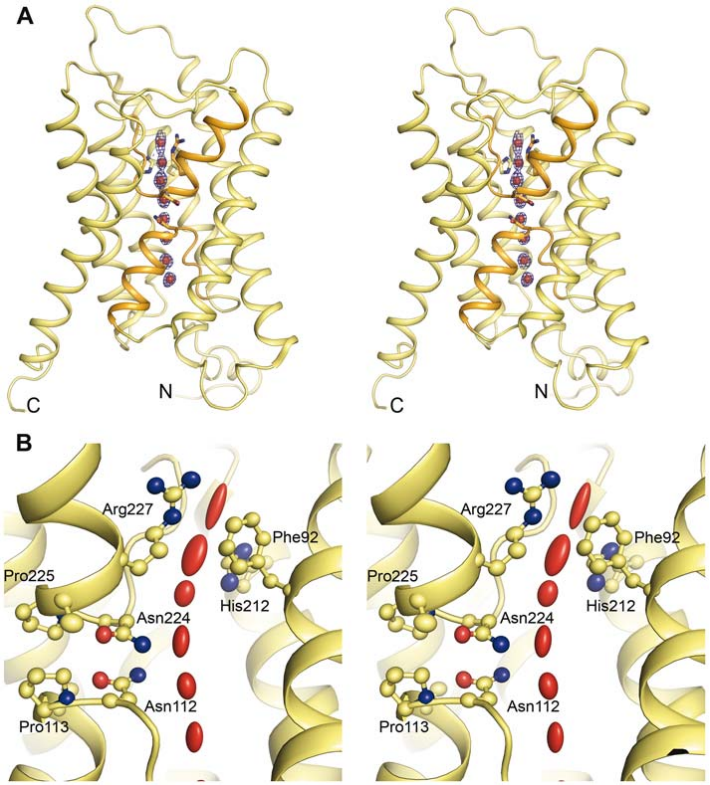
X-ray structure of Aqy1 at 1.15 Å resolution. (A) Stereo-view of a ribbon representation of the Aqy1 pore profile showing the overall structure. The two half-helices of loops B and E, which contain the aquaporin NPA signature motif, are highlighted in orange. (B) Stereo-view of ellipsoid representation of the anisotropic motions. Several water molecules are present within the channel (red) and have variable anisotropy. Carbon atoms are shown in yellow, nitrogen atoms in blue, and oxygen atoms in red.

To shed light on the unique functional attributes of yeast aquaporins we crystallized and solved the X-ray structure of Aqy1 from *P. pastoris* to 1.15 Å resolution (Table 1). As is common for aquaporins, Aqy1 crystallized as a tetrameric assembly in the I4 space group with one monomer per asymmetric unit, with crystals growing as stacked membrane bilayers having a gap of approximately 22 Å between the tetramer surfaces (Figure S1). In particular, the N and C termini do not take part in crystal formation and hence the conformation of these termini is not influenced by crystal packing. The tetramer consists of four independent water channels, in which each monomer has six transmembrane helices that adopt the typical “hourglass” fold [4],[17]–[20],[22],[23] (Figure 1A). Loops B and E insert into the membrane and together form a seventh transmembrane pseudo-helix containing the aspargine-proline-alanine (NPA) aquaporin signature motif near the center of the water channel. The water pore narrows on the extracellular side near the aromatic/arginine (ar/R) constriction region (Figure 1A), which functions as a selectivity filter.

**Table 1:** Data collection and refinement statistics.

Data Collection and Refinement	Statistic	Subcategory	Aqy1 (pH 3.5) Crystal 1	Aqy1 (pH 8.0) Crystal 2
**Data collection**		**Space group**	I4	I4
	**Cell dimensions**	***a*** **, ** ***b*** **, ** ***c*** ** (Å)**	91.4, 91.4, 80.8	90.9, 90.9, 80.6
		**Α, β, γ (°)**	90, 90, 90	90, 90, 90
		**Resolution (Å)**	1.15 (1.20–1.15)	1.40 (1.48–1.40)
		***R*** **_sym_**	8.0 (73.3)	8.8 (70.6)
		***I*** **/σ** ***I***	9.3 (2.2)	12.2 (1.8)
		**Completeness (%)**	94.7 (99.1)	100 (100)
		**Redundancy**	3.9 (3.2)	5.3 (3.4)
**Refinement**		**Resolution (Å)**	20.0–1.15	64.5–1.4
		***n*** ** Reflections**	111,455	61,170
		***R*** **_work_/** ***R*** **_free_**	14.0/16.5	16.2/18.1
		***R*** **_work_/** ***R*** **_free_ (** ***F*** **_0_>4σ)**	11.4/13.6	—
	***n*** ** Atoms**	**Protein**	2,073	2,080
		**β-OG**	120	80
		**Cl^−^**	3	2
		**Water**	143	133
	***B*** **-factors**	**Protein**	15.9	10.4
		**β-OG**	48.7	24.3
		**Cl^−^**	18.9	17.1
		**Water**	28.6	24.1
	**R.m.s. deviations**	**Bond lengths (Å)**	0.014	0.008
		**Bond angles (°)**	1.9	1.07

The structure was determined from two crystals obtained at different pHs. Values for the highest resolution shell are given in parenthesis. Abbreviations: R.m.s, root mean square.

The very high resolution of this structure made it possible to include anisotropic temperature factors during structural refinement. The anisotropic thermal motion is described by six anisotropic displacement parameters (ADPs), and the anisotropy of an atom is then defined as the ratio of the minimum and maximum eigenvalues of the 3×3 matrix formed by the ADPs. This definition means that the ratio is 1.0 for a perfectly isotropic (spherical) motion and decreases with increased anisotropic (nonspherical) motion. The anisotropic thermal motions of the atoms in the water pore are represented as ellipsoids in Figure 1B. It is apparent that eight water molecules are present within the channel, and their anisotropic motions align approximately parallel with the direction of water transport. In contrast, the side chain nitrogen atoms of Asn112 and Asn224, which belong to the dual NPA signature motif, show low anisotropy (0.6–0.8 for the nitrogen atoms compared to 0.3 for the closest water), and their movement is not synchronized with water molecules to which they hydrogen bond. Thus, at this resolution it is possible to visualize how the H-bond acceptor atoms of the dual NPA motif serve as rigid structural anchors, necessitating a specific orientation of water molecules as they pass and thereby helping to exclude the cotransport of protons [2]–[4]. Similarly, although water molecules near the aromatic/arginine constriction region show exceptionally large anisotropic motions, with anisotropy as low as 0.1, the side-chain atoms of Arg227 adopt a well ordered conformation with anisotropy in the range of 0.6–0.8 for the side chain (Figure 1B). This lack of flexibility of the side chain of Arg227 does not support the suggestion that dual conformations of the homologous arginine may gate water transport activity in AqpZ [25].

### Tyrosine Residue Blocks the Channel

**Figure 2:**
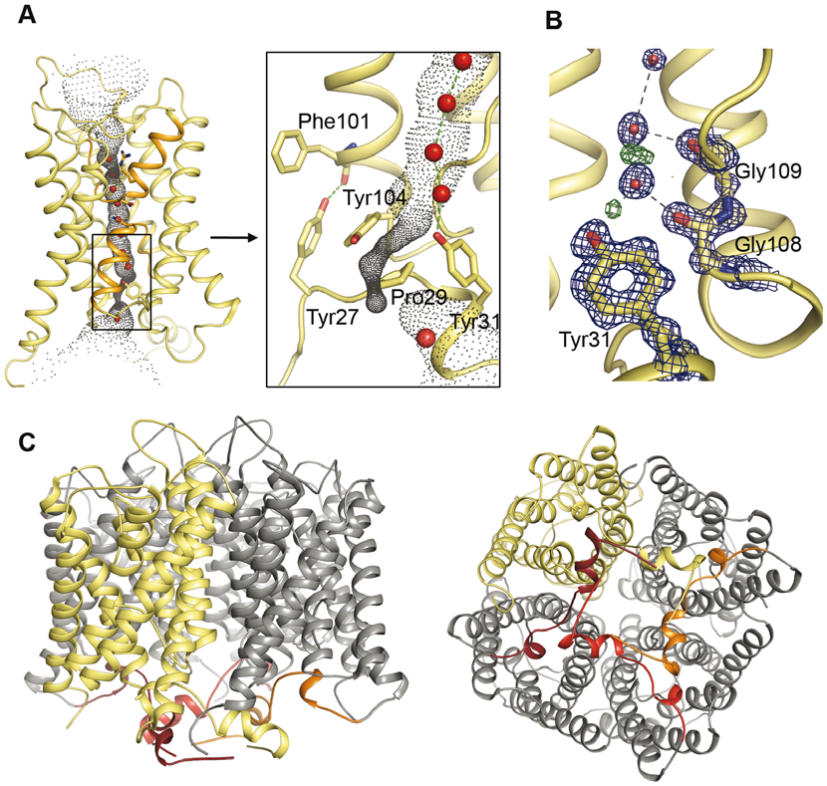
Aqy1 is closed on the cytoplasmic side by the N terminus. (A) A HOLE representation and an inset showing a close up view of the Aqy1 water channel constriction near Tyr31. (B) 2*F*_obs_−*F*_calc_ electron density map (blue, contoured at 2.0 σ) and *F*_obs_−*F*_calc_ electron density map (green, contoured at +2.2 σ) near Tyr31. Residual positive *F*_obs_−*F*_calc_ electron density reveals electrons associated with protons participating in hydrogen bond interactions. (C) The N termini from the four subunits of the tetramer twist around each other and form an N-terminal bundle positioned on the cytoplasmic side of the membrane.

**Figure 3:**
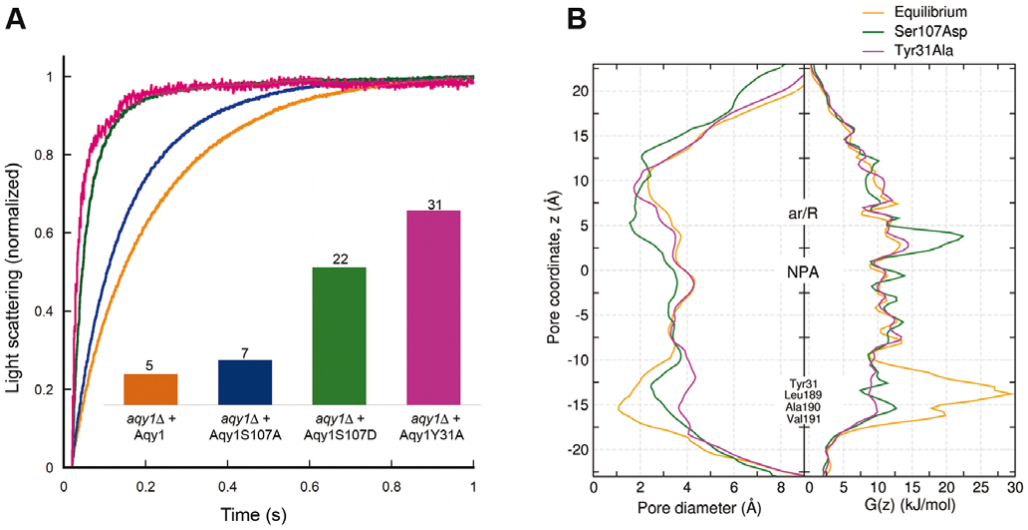
Proposed mechanism of Aqy1 regulation by phosphorylation. (A) Water transport activity measured by the change in light scattering of *P. pastoris* spheroplasts upon shrinkage caused by subjecting the cells to a hyperosmolar solution. Aqy1 (orange), Aqy1S107A (blue), Aqy1S107D (green), and Aqy1Y31A (magenta) were overproduced in *aqy1*Δ (endogenous AQY1 gene disrupted). The inset shows the rate constants (s^−1^) obtained by curve fitting of a single exponential function to 1 s of raw data. (B) Pore diameter and free energy profiles recovered from molecular dynamics simulations of Aqy1 (orange), Aqy1S107D (green), and Aqy1Y31A (magenta).

Strikingly, a functional role for the yeast aquaporin N terminus is implied by the structure of Aqy1, since the water channel is closed on the cytoplasmic side by conserved N-terminal residues (Figures 2 and S2). Specifically, the N terminus folds such that each Aqy1 protomer is intertwined with its neighbour within the tetramer via a helical bundle, which is stabilized by multiple hydrogen bond interactions (Figures 2C and S3). The presence of the bundle contrasts with all other reported aquaporin structures where primarily hydrophobic interactions stabilize the tetramer formation. Hydrogen bond from Tyr27 anchors the bundle of Aqy1 to the aquaporin scaffold and Pro29 introduces a kink (Figure 2A) allowing Tyr31 to insert into the water channel. The hydroxyl group of Tyr31 partakes in an exceptionally well ordered hydrogen bond network involving two water molecules and the backbone carbonyl oxygen atoms of Gly108 and Gly109, with residual *F*_obs_−*F*_calc_ electron density revealing the H-bond donor-acceptor relationships between these groups (Figure 2B). A HOLE [26] representation of the pore profile reveals how the water channel narrows to 0.8 Å in diameter near Tyr31, which is too small to allow the passage of water (Figure 3B). Moreover, when Tyr31 of Aqy1 is substituted by an alanine there is a 6-fold increase in water transport activity (Figure 3A). Equilibrium molecular dynamics simulations also establish the existence of an energetic barrier to water permeation of approximately 30 kJ/mol in this region (Figure 3B), confirming the closed nature of the channel. When Tyr31 is substituted with an alanine in silico the pore widens to a diameter larger than 2 Å, which reduces the free energy barrier to water permeation to less than 13 kJ/mol (Figure 3B), and water molecules enter the channel and fill the space left by Tyr31.

The blocking of the Aqy1 channel with a tyrosine residue is reminiscent of the situation found in mammalian AQP0 (Figure S4), which helps form thin junctions in the lens core of mammalian eyes. AQP0 has a water transport activity an order of magnitude lower than that typically found for other human aquaporins [27] and its structure has been solved in both a nonjunctional (open) form as well as in junctional (closed) form [20],[22],[28]. The closing of the pore is believed to be caused by proteolytic cleavage of AQP0 and also (to some extent) by the addition of calcium or changes in pH [29]. It has been suggested that subtle changes in the conformation of Tyr149 on the extracellular side may provide a mechanism whereby the water flux through the channel can be regulated [28],[30],[31].

### Aqy1 Is an N-Terminal Gated Aquaporin

A hallmark for yeast aquaporins is an extended N terminus. Whereas the N terminus of the aquaglyceroporin Fps1 from *S. cerevisiae* has been suggested to regulate the opening of the channel upon osmotic changes [11], the N terminus of the orthodox yeast aquaporins has not yet been assigned any regulatory function. To investigate the functional role of the N terminus of Aqy1, water conduction through Aqy1 was assayed using *P. pastoris* spheroplasts.

**Figure 4:**
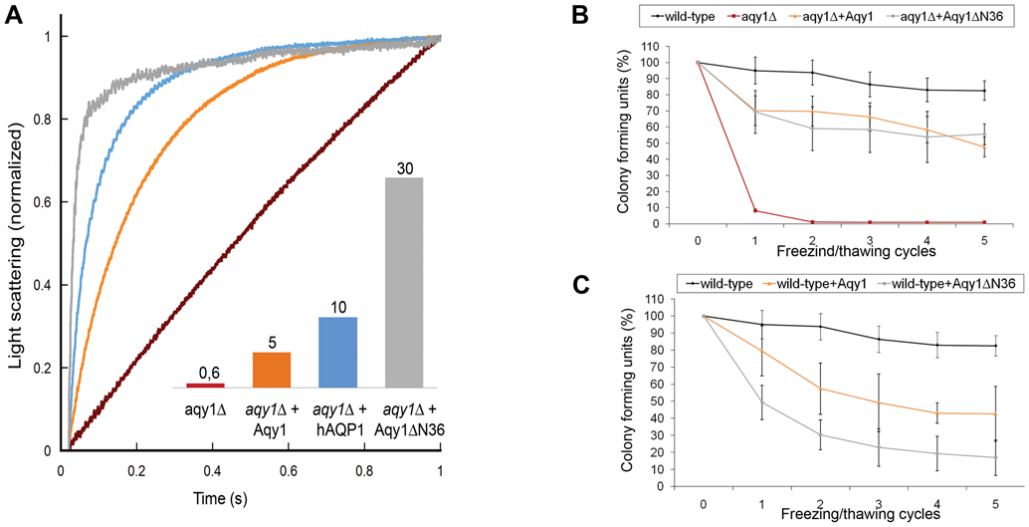
Water transport activity and growth assays on Aqy1. (A) Water transport activity measured by the change of light scattering of *P. pastoris* spheroplasts upon shrinkage caused by subjecting the cells to a hyperosmolar solution. Aqy1 (orange), hAQP1 (blue), and Aqy1ΔN36 (grey) were overproduced in the *aqy1*Δ-strain (red, endogenous *AQY1* gene disrupted). The inset shows the rate constants (s^−1^); the curves were smoothened for clarity. (B) Relative number of *P. pastoris* colony forming units (CFU) following multiple freeze/thaw cycles (*x*-axis) of the wild-type strain (black), *P. pastoris* with the *AQY1* gene disrupted (*aqy1*Δ) (red); *aqy1*Δ overproducing Aqy1 (orange) and Aqy1ΔN36 (grey). (C) Similar experiments as in (B) but using wild-type *P. pastoris* strain (black) and overproducing recombinant Aqy1 (orange) and Aqy1ΔN36 (grey). The recombinantly overproduced proteins negatively impact survival, with Aqy1ΔN36 having a significantly larger effect.

Water transport activity measurements showed that the presence of wild-type Aqy1 in the *P. pastoris* membrane increases water permittivity. In particular, the transport rates for spheroplasts overexpressing Aqy1 were eight times faster than for the *aqy1*Δ-strain of *P. pastoris* (an engineered strain with the gene encoding Aqy1 disrupted). Thus, although the crystal structure of Aqy1 is closed, it is an active aquaporin. However, when water transport activities were compared for strains overexpressing wild-type Aqy1 and overexpressing Aqy1 with a truncated N terminus (Aqy1ΔN36, 36 N-terminal residues deleted), it was apparent that deleting the N terminus increased the water transport activity by a factor of six (Figure 4A). Since full-length Aqy1 is functionally active yet structurally closed, and its activity is increased by deleting the N terminus, we conclude that it is gated by its N terminus. Moreover, the high degree of conservation of Pro29, Tyr31, Gly108, and Gly109 implies that the proposed gating mechanism is prevalent across other yeast species (Figure S2).

### Aqy1 Enhances Survival upon Freezing

Aquaporin expression in yeast, particularly in *S. cerevisiae,* is strongly correlated with freeze tolerance [12]. Deletion of the gene encoding Aqy1 in *P. pastoris* (*aqy1*Δ) also leads to a markedly reduced tolerance of multiple cycles of rapid freezing in liquid nitrogen followed by thawing (Figure 4B). The ability of *aqy1*Δ to survive repeated freeze/thaw cycles is recovered when either Aqy1 or a truncated form Aqy1ΔN36 is overproduced (Figure 4B). Surprisingly, there is no obvious advantage when reintroducing the *AQY1* gene over introducing the *AQY1*Δ*N36* gene. Nevertheless, overproduction of either protein (Aqy1 and Aqy1ΔN36) in the wild-type strain, for which endogenous Aqy1 is already present, did show detrimental effects on freeze/thaw survival for the permanently open Aqy1ΔN36 constructs (Figure 4C). These observations may be reconciled by accepting that (in this context) the survival benefits resulting from having at least one aquaporin present in the membrane masks the potential disadvantages of this aquaporin being permanently open. Taken together, these findings establish that water conduction through Aqy1 greatly enhances the survival of *P. pastoris* during rapid freezing, yet the effect is delicately balanced as the introduction of a permanently open aquaporin actually reduces the chances of survival. Thus, Aqy1 gating appears to mediate a compromise by balancing these conflicting effects by increasing water flux through the cell membrane in some contexts, and reducing it in others.

### Putative Phosphorylation Site Triggers Channel Opening

**Figure 5:**
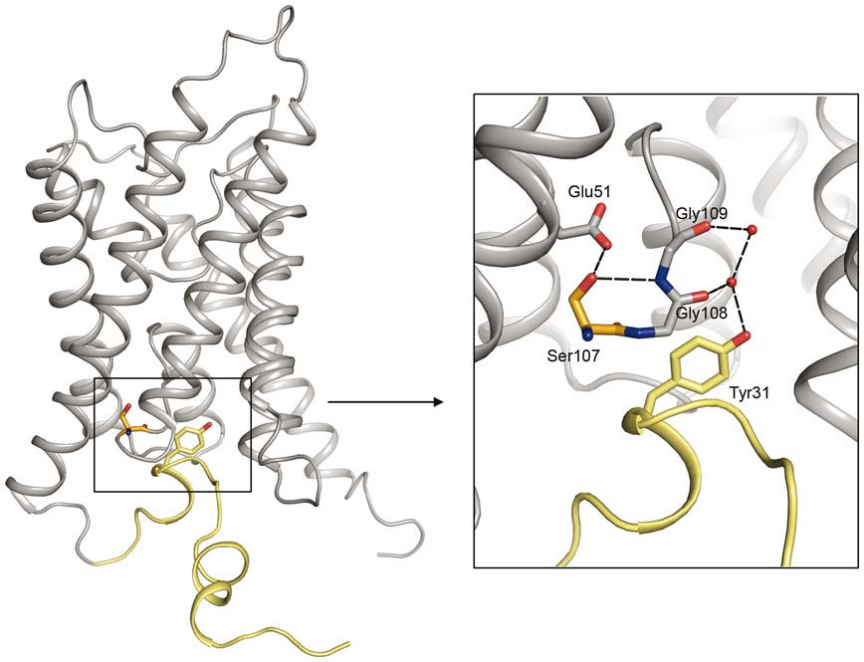
Putative phosphorylation site of Aqy1. Ribbon representation illustrating the location and close up view of the putative phosphorylation site Ser107. Aqy1 is shown in grey with its N terminus in yellow. The inset shows the hydrogen bond network in black.

Channel gating facilitates a rapid response to external stimuli when other regulatory mechanisms, such as transcriptional regulation or trafficking, are too slow. Since the gated plant plasma membrane aquaporin SoPIP2;1 is believed to be regulated by phosphorylation [32], we investigated the effects of mutation of candidate Aqy1 phosphorylation sites. Water transport assays in spheroplasts reveal a significant increase in water transport activity when Ser107 of Aqy1 was substituted by aspartate (where the aspartate mimics a putative phosphorylation event), yet water transport rates comparable to the wild type were recovered when it was substituted by an alanine (Figure 3A). Ser107 lies within a consensus phosphorylation site (according to NetPhos 2.0 Server [33]) situated near the pore channel and is involved in an important network of hydrogen bonds involving Tyr31 (Figure 5).

**Figure 6:**
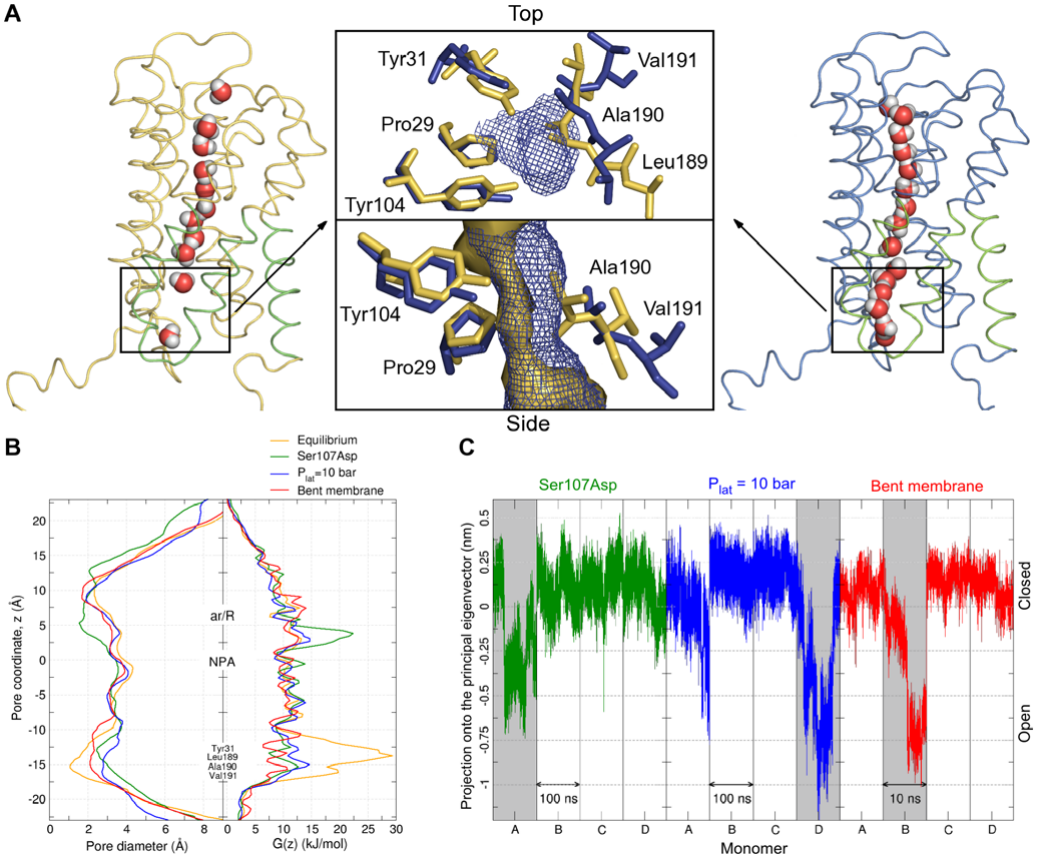
Molecular dynamics simulations of the two putative gating mechanism, phosphorylation and mechanosensitivity. (A) Snapshots of the closed (left) and the open (right) conformations showing how a water column is established in a similar way upon opening, during either simulations of the mutant Aqy1S107D, or increasing the lateral pressure to 10 bar, or bending the membrane towards the cytoplasmic side (the region highlighted in green was considered for the principal component analysis mentioned in the text). Top and side views of the region indicated by the dashed squares are shown in the middle panel: The pore surface is shown for both the closed (yellow) and the open (blue) conformations. The channel widens after a local rearrangement of residues Leu189, Ala190, and Val191, and this motion is observed for all three simulations. (B) Pore diameter profiles (left), showing the widening of the channel near the Tyr31 region for simulations with the mutated Aqy1S107D (green), with an increased lateral pressure (blue), or with a bent membrane (red), compared with the control simulation (yellow). Free energy profiles (right), showing a substantial reduction in the energetic barrier for water permeation in the monomers that opened during the simulations. (C) Projections of nonequilibrium trajectories onto the principal eigenvector obtained by principal component analysis. Transitions are observed upon opening for the monomer A in the left panel (Aqy1S107D), D in the middle panel (P_lat_ = 10 bar), and B in the right panel (bent membrane).

Molecular dynamics simulations of S107D Aqy1 mutant also show a widening of the pore near these residues, which increases to a diameter larger than 2 Å, and a corresponding reduction in the free energy barrier to water permeation to less than 13 kJ/mol (Figure 3B). In these simulations water molecules establish a single-file water column between Pro29, Tyr31, Tyr104, Leu189, Ala190, and Val191, after a local rearrangement of the latter three residues, which are located in the lower part of helix 4 towards loop D (Figure 6A). In contrast, simulations of the Y31A Aqy1 mutant allowed water molecules enter the channel, filling the space left by Tyr31. Thus both molecular dynamics simulations and functional data support the suggestion that Ser107 is a putative phosphorylation site, and that it induces an opening of the pore upon phosphorylation.

### Regulation by Mechanosensitivity

A putative mechanism of Aqy1 phosphorylation-regulated gating could explain the apparently low water transport activity of wild-type Aqy1 in *P. pastoris* spheroplasts (Figure 4A). Nevertheless, this idea does not explain why Aqy1 appears to have higher water-transport activity when purified and reconstituted into proteoliposomes than it has in its native membrane. In particular, when both Aqy1 and Aqy1ΔN36 (N-terminal truncated form) are reconstituted into proteoliposomes their water transport activities are almost indistinguishable (measured rate constants of 12.9 s^−1^ and 10.9 s^−1^, respectively; Figure S5), yet Aqy1 has a water transport activity of only one-sixth of that found for Aqy1ΔN36 in *P. pastoris* spheroplasts (Figure 4A). A clue hinting at an explanation for this apparent paradox is provided by the unique global topology of the Aqy1 tetramer, which has the N termini intertwined in a helical bundle (Figure 2C) in a manner that is reminiscent of the arrangement found for the mechanosensitive gated ion channel MscL from *Mycobacterium tuberculosis* (Figure S6) [34]. Mechanosensitivity has also been demonstrated for aquaporins found in plant roots [35]. Should Aqy1 be a mechanosensitive channel then this could accommodate the observation that it is more active in proteoliposomes than in its native membrane, because these proteoliposome vesicles are highly curved (120–130 nm in diameter; compared to spheroplasts with a diameter of 1–5 µm). Increased membrane curvature can activate mechanosensitive channels, as could destabilisation effects owing to the removal of native lipids.

To test this hypothesis, nonequilibrium molecular dynamics simulations of Aqy1 were performed in a solvated lipid bilayer being subject to external mechanical stress, either by increasing the lateral pressure up to 10 bar, or by bending the membrane towards the cytoplasmic side (Figure S7). Spontaneous opening events of one monomer in both simulations were observed (Figure 6A) with the pore diameter near Tyr31 widening from 0.8 Å in the crystal conformation to values larger than 2 Å in both simulations (Figure 6B). In addition, the energetic barrier for the water permeation dropped substantially compared to the control simulations in which no opening was observed (Figure 6B).

## Discussion

Since all unicellular life forms have a high surface-to-volume ratio, the intrinsic rate of water flow across their cell membranes was not likely to be rate limiting. Thus, the discovery of aquaporins in yeast was unexpected. In this study we demonstrate at least one physiological role for the aquaporin Aqy1 from *P. pastoris*, which imparts freeze tolerance to the host. Specifically, deletion of the gene causes a freeze-sensitive phenotype that can be remedied by reintroduction of the *P. pastoris AQY1* gene or the N-terminal truncated form of the *AQY1* gene (Figure 4A). In contrast, overproduction of the N-terminal truncated form of Aqy1 in the wild-type strain of *P. pastoris* results in a detrimental effect on freeze/thaw survival. Thus, the possibility to open and close the N terminus seems to be optimal for the survival of cells in freezing environments.

**Figure 7:**
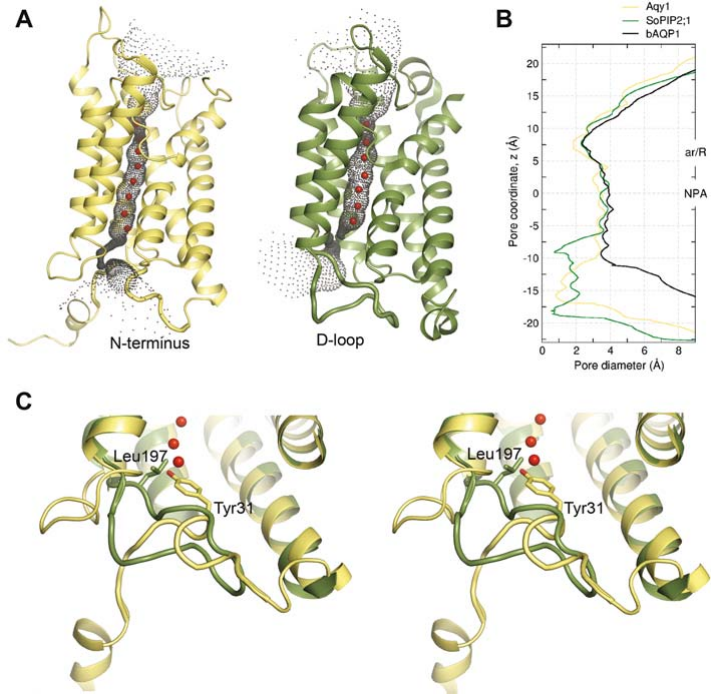
Structure comparisons of closed aquaporins from *P. pastoris* and spinach. (A) Comparison of the Aqy1 from *P. pastoris* (left panel) with the spinach aquaporin SoPIP2;1 (right panel) shown as HOLE representation. The pore is occluded by the N terminus in Aqy1, and by loop D in SoPIP2;1. (B) The pore diameter of closed Aqy1 (yellow), closed SoPIP2;1 (green), and constitutively open bAQP1 (black) structures calculated with program HOLE. (C) Stereo-view of the superposition of SoPIP2;1 (green) onto Aqy1 (yellow). Residues identified in the narrowest parts of the pores of the respective aquaporin are shown in stick representation.

Although yeast aquaporins have been extensively studied, there are no reports concerning the gating of orthodox yeast aquaporins. Nevertheless, Soveral et al. suggest that the water transport activity of Aqy1 from *S. cerevisiae* can be regulated by membrane tension [36], implicating gating. It has also been established that the aquaglyceroporin Fps1 of *S. cerevisiae* is rapidly regulated by osmolarity [13], which also implicates gating. The most conclusive functional evidence for aquaporin gating concerns the plant plasma membrane aquaporins [37]. In that case the water channel is closed by a unique conformation of loop D, which folds over the cytoplasmic entrance and blocks the channel (Figure 7) [23]. In contrast, our crystal structure of Aqy1, captured in a closed conformational state, shows that a tyrosine residue from the N terminus enters the pore (Figure 7) and blocks the opening of the channel by hydrogen bonding to a water molecule within the pore. According to sequence alignment, the N termini of aquaporins from yeast are well conserved (Figure S2), indicating that other yeast aquaporins may also share this N-terminal gating mechanism. In particular, the aquaporin from the fungal pathogen of humans *C. albicans* has all important amino acids conserved, suggesting that *C. albicans* aquaporin is gated in a similar manner as Aqy1 from *P. pastoris*.

Gated ion channels [38] participate in rapid physiological responses such as signal propagation. Likewise, the gated plant aquaporin SoPIP2;1 responds rapidly to cellular acidification as a result of flooding [6]. Our results show that Aqy1 is essential for yeast survival during rapid freezing and thawing, and for organisms of 1 to 10 µm in diameter this can occur within microseconds. Thus the survival benefits inferred by Aqy1 imply that channel opening must either be extremely rapid or that there are constitutively open channels in vivo. Since both the Aqy1S107D and N-terminal deleted Aqy1 (Aqy1ΔN36) mutants show considerably higher water transport activity than wild-type Aqy1 (Figures 3A and 4A), it is strikingly inefficient to express large quantities of Aqy1 in vivo, which has relatively poor water transport activity. From this perspective we argue that the observed host survival benefits arise from Aqy1 being quickly opened in response to rapid freezing.

pH-triggered opening, which is known to close SoPIP2;1 [6], can be excluded as a possible mechanism for Aqy1 since high-resolution structures recovered at both acidic and basic pH are identical, with a root mean square deviation of only 0.08 Å for 248 Cα atoms (Figure S8). Thus mechanosensitive gating emerges as a plausible mechanism. This possibility is supported by molecular dynamic simulations, which show how Aqy1 can be regulated by both the surface tension and membrane curvature (Figure 6). An intriguing question for the mechanism of mechanosensitivity is how a mechanical signal is transmitted from the membrane to the gate of the channel in the cytoplasmic portion of helix 4, which is not in direct contact with the membrane. To address this question, a principal component analysis of the backbone atoms of the cytoplasmic halves of helices four, five, six, and loop D was carried out. Projections of both membrane-mediated stress trajectories onto the principal eigenvector show an abrupt transition for the monomers in which an opening takes place (Figure 6C), suggesting that external forces triggering gating are transmitted from the lipid membrane to Leu189, Ala190, and Val191 via coupled movements of the helices four, five, and six, the latter being in direct contact with the membrane. To investigate the causal relation between this global conformational change and channel opening, the position along this principal eigenvector was artificially driven from the “closed” to the “open” conformation in an additional set of simulations, leading to reproducible channel opening in 14 of 16 cases (Figure S9).

It is intriguing to consider how the two putative modes of regulation, by both phosphorylation of a serine residue or changes in the surface tension and curvature of the membrane (Figures 3A and 6B), may relate to each other. Strikingly, the opening transition for the Aqy1S107D simulation, which mimics a phosphorylated S107, involves similar movements of residues Leu189, Ala190, and Val191 as in the simulations with the membrane being subject to external mechanical stress (Figure 6A). These similarities are revealed in a principal component analysis of the Aqy1S107D trajectory, where the projection onto the first eigenvector, upon opening, drops in a manner similar to simulations with induced membrane stress (Figure 6C). These findings disclose that both phosphorylation and external membrane-mediated mechanical stress may induce the same opening mechanism, and suggest how mechanical stimuli are transmitted from the membrane to the gate of the channel. Post-translational regulation of Aqy1 by phosphorylation may also be exploited in other physiological contexts, when less rapid changes in water transport activity are required. Hence, phosphorylation may fine-tune the water flux during normal conditions of growth, whereas mechanosensitive gating could provide a rapid pressure valve in response to unexpected shocks.

Rapid freezing or thawing and sudden osmotic changes are frequently encountered by micro-organisms [8]. For example, actions of warm blooded animals in cold environments, such as breathing, coughing, walking, and foraging, can expose micro-organisms to large temperature shocks. Likewise, dramatic changes in osmolarity arise when microbes encounter ripe fruit or rainwater. Indeed, the aquaglyceroporin Fps1 of *S. cerevisiae* is suggested to be rapidly opened when the host is exposed to sudden osmotic changes [11]. Thus the evolution of gated aquaporins and aquaglyceroporins would provide an economic solution to numerous stresses associated with rapidly changing environments, aiding the organism's quest to adapt and survive.

## Methods

### Yeast Strains and Cloning

The *AQY1*-deficient strain was created by insertion of *HIS4* into *AQY1*, disrupting the *AQY1* ORF. The *S. cerevisiae HIS4*-promoter and ORF were amplified by PCR (primers A and B [Table S1]). PstI (New England BioLabs) linearized pPICZαB-*AQY1*-vector and PstI-digested PCR product were ligated and transformed into *Escherichia coli*. The created *AQY1-HIS4-AQY1* deletion construct was amplified by PCR (primers C and D) (Table S1) and transformed into *P. pastoris* GS115-*his4* according to Invitrogen's manual [39]. Transformants were selected for histidine prototrophy and disruption of *AQY1* was confirmed by PCR. His_6_-tagged constructs of Aqy1 and Aqy1ΔN36, i.e. Aqy1 lacking the soluble part of the N terminus (first 36 residues), were prepared from genomic DNA by PCR using primers E and F (Table S1), respectively. Antisense-primer G was used for fusing a C-terminal His_6_-tags to the ORFs. PCR-products and pPICZαB-vector (Invitrogen) were digested with EcoRI/XbaI (New England BioLabs) and ligated, creating pPICZαB-*AQY1*-His_6_ and pPICZαB-*AQY1*ΔN36-His_6_, respectively. The vectors were linearized using PmeI and transformed into wild-type and *AQY1*-deficient *P. pastoris* strains. Transformants were selected for on Zeocin-containing plates. Point mutations of Aqy1 (Y31A, S107D, and S107A) were created by site-directed mutagenesis using the Quikchange II- and Quikchange Lightning-kits (Stratagene).

### Protein Expression and Purification

All cultures were grown in a 3 l bioreactor and the protein expression was induced by methanol according to standard procedure (Invitrogen). Cells were lysed using X-press equipment (AB Biox) and resuspended in Breaking Buffer (50 mM KH_2_PO_4_/K_2_HPO_4_-buffer, [pH 7.5], 5% glycerol). After removal of cell debris by centrifugation at 15,000*g*, 20 min, 4°C, the membrane fraction was collected by centrifugation at 138,000*g*, 90 min, 4°C and washed with breaking buffer. The membranes were solubilized with beta-octylglucopyranoside (5% β-OG, 20 mM Tris [pH 8.0], 100 mM NaCl, 20% glycerol, 0.5 mM EDTA). Unsolubilized material was removed by centrifugation at 15,000*g*, 20 min, 4°C. The His-tagged protein constructs were purified for the liposome assay (Figure S5) by immobilized metal ion affinity chromatography (IMAC) using Ni-NTA resin (Qiagen) and eluted with 500 mM imidazole in 20 mM Tris, (pH 8.0), 100 mM NaCl, 20% glycerol, 1% β-OG. Further purification was performed by size-exclusion chromatography using a Superdex200 column (GE Healthcare). The purity of the samples was verified by SDS-PAGE and MS.

For crystallization, nontagged, endogenously expressed Aqy1 was used. The protein was purified using ion exchange column ResourceQ (GE Healthcare) and size exclusion chromatography on Superdex200 (GE Healthcare) according to Nyblom et al. [40].

### Crystallization and Data Collection

The protein was concentrated to 9 mg/ml and crystallization trials were set up using the hanging drop vapour diffusion method at 4°C, with drops containing equal volumes of protein- and reservoir-solution. Crystals appeared in two different conditions, at different pH. Crystal1 (Figure S10) grew in 28% PEG400, 100 mM Na-citrate (pH 3.5), 200 mM Li_2_SO_4_, while Crystal2 (Figure S10) grew in 33% PEG400, 100 mM Tris (pH 8.0), 100 mM NaCl, 100 mM CdCl_2._ The crystals were frozen in liquid nitrogen and diffraction data were collected under cryoconditions at 0.873 Å on beamline ID23-2 at the European Synchrotron Radiation Facilities (ESRF, Grenoble, France) and at 1.04 Å at MaxLAB (Lund, Sweden).

### Structure Determination

High- and low-resolution data for Crystal1 (1.15 Å) were processed and scaled using XDS [41], and a molecular replacement solution was found using Phaser [42] with bovine AQP1 (Protein Data Bank [PDB, http://www.rcsb.org/pdb] accession code, 1J4N) as search model. Initial model building was carried out with Arp/Warp [43], and further model building was performed in COOT [44]. To verify the solution, unbiased composite omit maps were calculated using CNS [45]. Initial refinement was made using Refmac5 [46], while SHELXL [47] was used in the last stage of refinement. The model was refined anisotropically (with exception of β-OG, which was refined as rigid body) and hydrogen-atoms were added to protein atoms as a riding model. No hydrogen atoms were added along the water-channel to allow for the correct observation of difference density. In the Ramachandran [48] plot 91.8% and 8.2% of the residues fell in favored and allowed regions respectively. Data from Crystal2 (1.4 Å) were processed using MOSFLM [49]. Phaser was used to find a molecular replacement solution using bovine AQP1 (PDB accession code, 1J4N). Model building was carried out using Arp/Warp and COOT. The refinement, including simulated annealing with omit map calculations and restrained anisotropic refinement, was performed using CNS and Refmac5. In the Ramachandran plot 92.7% and 7.3% of the residues fell in favored and allowed regions respectively. Data collection and refinement statistics are shown in Table 1.

### Functional Assays

For freezing and thawing experiments *P. pastoris* cells were grown in BMMY (Invitrogen), starting at OD_600_ = 1, for 24 h at 30°C for induction of protein production. In total, five cycles of freezing aliquots of 1 ml, OD_600_ = 5×10^−4^, in liquid nitrogen and subsequential thawing in a water bath at 30°C were followed by spreading aliquots on YPD plates.

The spheroplast water transport assays is based on the shrinkage of *P. pastoris* cells with a destabilized cell wall after subjection to a hyperosmolal solution. *P. pastoris* spheroplasts [50] were created in biological triplicates by inducing recombinant protein production through growth in BMMY for 22 h, starting at OD_600_ = 1. The cells were harvested and incubated with TE-buffer (100 mM Tris [pH 8.0], 1 mM EDTA) and 0.5% β-mercaptoethanol for 1.5 h to destabilize the cell wall. Subsequently cells were washed with 1.2 M Sorbitol, 20 mM MES (pH 6.5), and resuspended to final OD_600_ = 5. The shrinkage upon mixture was observed by light scattering at 90° angle in a stopped-flow apparatus (Biologic Instruments Inc.) at 435 nm.

The water transport assay is based on the shrinkage of proteoliposomes after subjection to a hyperosmolal solution. Liposomes were prepared according to Nyblom et al. [40]. In brief, liposomes were created from *E. coli* polar lipid extract (Avanti) by sonication and the protein was reconstituted using β-octylglucopyranoside, which was subsequently removed by dilution. The shrinkage was observed by measuring light scattering at a 90° angle in a stopped-flow apparatus (Biologic Instruments Inc.) at 480 nm. Rate constants were obtained by curve-fitting to 1 s of raw data using the equation: *y* = *A1*×*exp*(−*k*×(*t*−*td*))+*A2* (*A1* = amplitude, *k* = rate constant, *A2* = vertical offset, *td* = constant time delay [s]).

Western blot analysis of the spheroplasts used for the water transport assay was performed according to ECL Plus manual (GE Healthcare) with His_6_ monoclonal primary antibody (Clontech) on the membrane fraction of the constructs. The cells were lysed with glass beads in cell resuspension buffer CRB (20 mM Tris [pH 8.0], 100 mM NaCl, 0.5 mM EDTA, 5% glycerol) using a FastPrep-24 (M.P. Biomedicals). Cell debris was removed by centrifugation at 10,000*g*, 30 min, 4°C, and the membrane fraction was collected from the supernatant by centrifugation at 100,000*g*, 90 min at 4°C. The membranes were dissolved in CRB containing 5% β-OG and the concentration was adjusted to equal Abs_260 nm_ values with CRB+β-OG to ensure same amounts of membrane were loaded (Figure S11).

### Molecular Dynamics Simulations

Molecular dynamics simulations (Text S1) were carried out using the GROMACS simulation software [51],[52], with the Aqy1 tetramer embedded in a solvated POPE bilayer (Figure S7). Five different simulations were carried out: first, under equilibrium conditions, without surface tension (I); second, mutating Ser107 into Asp, mimicking a putative phosphorylated state (II); third, mutating Tyr31 into Ala (III); fourth, inducing a surface tension onto the membrane (IV), and fifth, bending the membrane towards the cytoplasmic side (V). The simulation boxes contain the protein [53],[54], POPE lipids [55], SPC water molecules [56], and chloride ions to neutralize the system. Both the temperature and pressure were kept constant by coupling the system to an external bath at a temperature of 300 K and a pressure of 1 bar [57], respectively. To induce a surface tension in simulation IV, the pressure in the *xy* plane (parallel to the membrane surface) was increased to 10 bar. To bend the membrane in simulation V, lipids at distances larger than 5 nm from the center of the tetramer were pushed along the *z* coordinate, exerting an external force that resulted in an acceleration of 0.01 nmps^−2^. A force of equal magnitude and in the opposite direction was exerted on the tetramer for compensation. The system was equilibrated for 1 ns by maintaining the coordinates of the protein harmonically restrained. The simulation length was 100 ns and 10 ns for the simulations I to IV and V, respectively. Pore diameter profiles were obtained with the HOLE software [26]. Free energy profiles were computed using the formula *G*(*z*) = −*k*_B_ *Tln*〈*n*(*z*)〉, where *k*_B_ is the Boltzmann constant, *T* is the temperature, and 〈*n*(*z*)〉 is the water density as a function of the pore coordinate. A principal component analysis, consisting of the calculation and diagonalization of the covariance matrix for the coordinates of the backbone atoms of the lower part of helices four, five and six, and loop D [58], was carried using GROMACS.

### Accession Codes

Protein Data Bank (http://www.rcsb.org/pdb) coordinates for Aqy1 (low and high pH structures) have been deposited with accession codes 2W2E and 2W1P.

## Supporting Information

Figure S1 **Crystal packing of Aqy1.** (A) shows the *xz*/*yz* plane, and (B) shows the *xy* plane of the crystal. The gap between the tetramer surfaces is approximately 22 Å.
(1.35 MB TIF)

Figure S2 **Sequence alignment of orthodox aquaporins from different yeast species.** The sequence numbering corresponds to Aqy1 from *P. pastoris*. The residues marked yellow are potentially involved in the gating mechanism. The C-terminal parts of the sequences are not shown. The alignment was made with ClustalW.
(0.24 MB TIF)

Figure S3 **Stereo-view of the N-terminal helical bundle.** Aqy1 has an N-terminal extension of 34 residues compared with the human homologue AQP1. This extension forms into two short α-helices. The N termini from the four subunits of the tetramer twist around each other and form an N-terminal bundle positioned on the cytoplasmic side of the membrane. The interactions between the N termini are shown in stereo-view. In addition to the interactions shown in this figure, the N termini also interact with loop D and the C terminus of the neighbouring subunits.
(0.93 MB TIF)

Figure S4 **The water channels of yeast Aqy1 and mammalian AQP0 are blocked in similar fashion by a tyrosine residue.** Tyr31 in Aqy1 (yellow) and Tyr149 in AQP0 (light brown, PDB code 2b6o) are shown as sticks. Water molecules found within the channel of Aqy1 are shown as orange spheres, whereas light brown spheres show water molecules found in the pore of AQP0. For clarity, only aspargine residues from the NPA regions are shown in sticks representation.
(0.86 MB TIF)

Figure S5 **Water transport assay using light scattering spectroscopy and proteoliposomes.** Change in light scattering (normalized) is plotted against time of Aqy1-his (green) and Aqy1ΔN36-his (blue) protein reconstituted into liposomes upon 1:1 mixing with an 826-mOsm external hyperosmotic sorbitol solution, compared to a control of empty liposomes (red). The inset shows the physical data obtained from curve fitting to a double exponential function. Data were derived from an average of ten traces and the rate constant values (*k*) of the dominant fraction were used for calculation of the permeability coefficients *P*_f_. Liposome sizes were determined by dynamic light scattering, the first cumulant value is given. Both constructs appear to be fully open when reconstituted into liposomes. In contrast, wild-type Aqy1 has a water-transport activity only one-sixth of that observed for Aqy1ΔN36 in the *P. pastoris* spheroplast assay (Figure 3A). The increase in activity of wild-type Aqy1 when reconstituted into proteoliposomes may be due to their high curvature triggering mechanosensitive opening of the channel (Figure 6).
(0.29 MB TIF)

Figure S6 **Side by side comparison of the structure of Aqy1 and MscL.** The N-terminal bundle of Aqy1 (left) is reminiscent of the N-terminal bundle observed in the structure of the mechanosensitive gated ion channel MscL (right). Both structures are shown parallel with membrane.
(2.20 MB TIF)

Figure S7 **Molecular dynamics simulations of Aqy1.** Simulation boxes showing the tetramer (blue, orange, red, and cyan), fully embedded in a lipid bilayer (yellow head groups and green tails) and solvated by water (blue, white), for the simulations without (left and middle panels, showing side and top views, respectively) and with (right panel) an induced bending of the membrane.
(2.54 MB TIF)

Figure S8 **Superposition of the Aqy1 structures crystallized at pH 3.5 and pH 8.0.** The crystal structures of the *P. pastoris* Aqy1 crystallized at pH 3.5 (yellow) and pH 8 (green) respectively show identical closed conformation. The RMS deviation is 0.08 Å for 248 Cα atoms, and 0.4 Å using 1,969 atoms of the structure. This is a strong indication that Aqy1 is not regulated by pH.
(0.78 MB TIF)

Figure S9 **Essential dynamics simulations of Aqy1.** (A) Projections of all trajectories onto the principal eigenvector found in the PCA analysis (see Figure 6C). Initially, for each monomer the driving velocity was 1 nm/ns (black lines). Then, after 0.8 ns (red), 0.9 ns (green), 1.0 ns (blue), and 1.1 ns (orange), the driving velocity was set to 0 nm/ns. (B) Averaged pore diameter profiles for each one of the four monomers, during the second part of the simulation (driving velocity equals 0 nm/ns), after 0.8 ns (red), 0.9 ns (green), 1.0 ns (blue), and 1.1 ns (orange). The pore widens near Tyr31 to values larger than 2 Å for 14 of the 16 cases, indicating that the collective coordinate represented by the first eigenvector is indeed responsible of the gating conformational change, and that the opening motions taking place at the gate of the pore are reproducible separately in the four monomers.
(0.68 MB TIF)

Figure S10 **Crystals of Aqy1 from pH 3.5 and 8.0.** (A) shows Crystal 1 (pH 3.5, 1.15 Å) and (B) shows Crystal2 (pH 8.0, 1.4 Å).
(1.72 MB TIF)

Figure S11 **Western blot of** ***P. pastoris*** **membrane fractions used for the spheroplast water transport assay showing the expression levels of the respective proteins.** The same amount of membrane was loaded for all constructs, except for hAQP1, for which 30× as much membrane was loaded to achieve a comparable signal. All other proteins are expressed at comparable levels. Although the S107D Aqy1 mutant may be expressed at slightly lower levels than the other Aqy1 constructs, this mutant shows a significantly increased water-transport activity in the *P. pastoris* spheroplast assay when compared with overproduced wild-type Aqy1. The major conclusions of the spheroplast assay (Figures 3A and 4A), that wild-type Aqy1 has a significantly lower water-transport activity than the other Aqy1 constructs, is thus unaffected by normalization against the protein yield.
(0.05 MB TIF)

Table S1 **Primers used for cloning.** Nucleic acid sequences for primers used for creating the *AQY1* deficient *P. pastoris* strain as well as strains overexpressing Aqy1 and mutants of Aqy1.
(0.03 MB DOC)

Text S1 **Supplementary information on molecular dynamics simulations.**
(0.07 MB DOC)

## Abbreviations

Aqy: aquaporin from yeast
AQP: aquaporin
NPA: aspargine-proline-alanine
