# Supplementary figures and images for: Crystal Structure of a Yeast Aquaporin at 1.15 Å Reveals a Novel Gating Mechanism
Source: PLoS Biol. 2009 Jun 16;7(6):e1000130. doi: 10.1371/journal.pbio.1000130 (PMC2688079; doi:10.1371/journal.pbio.1000130)

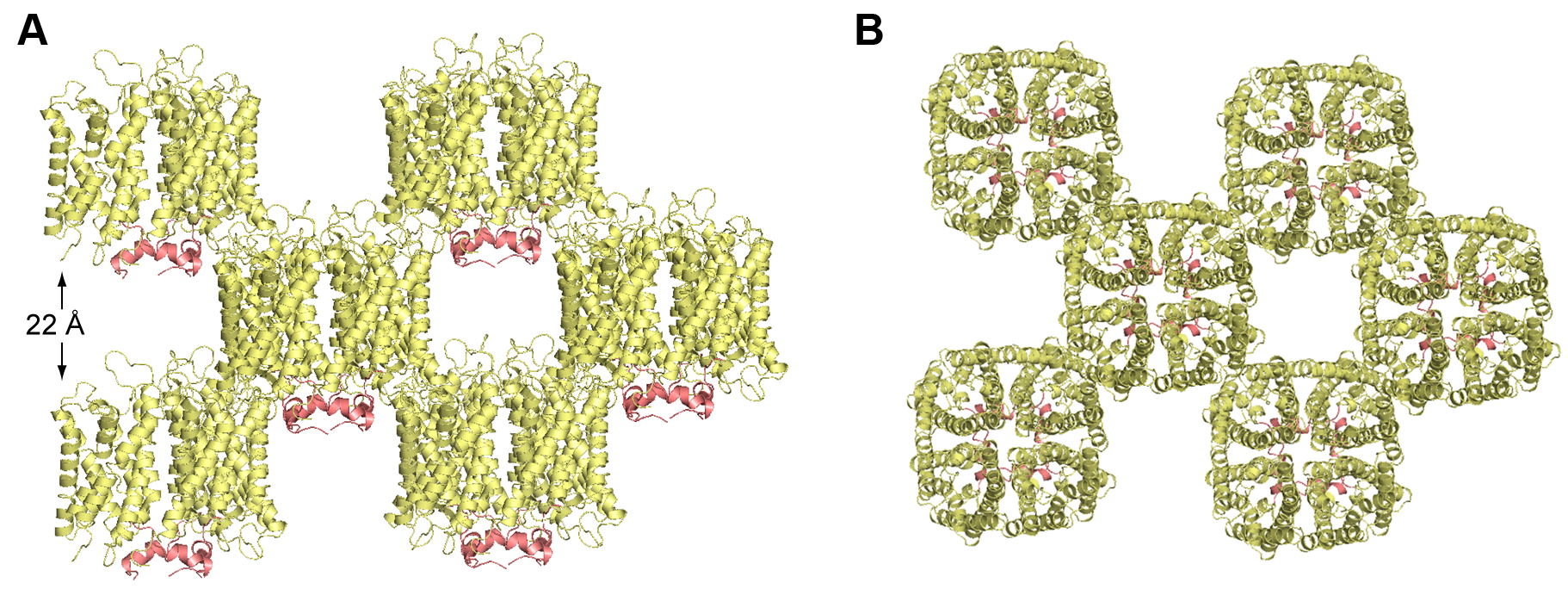

Supplement: Figure S1 — Crystal packing of Aqy1. (A) shows the xz/yz plane, and (B) shows the xy plane of the crystal. The gap between the tetramer surfaces is approximately 22 Å. (1.35 MB TIF) [file pbio.1000130.s001.tif]

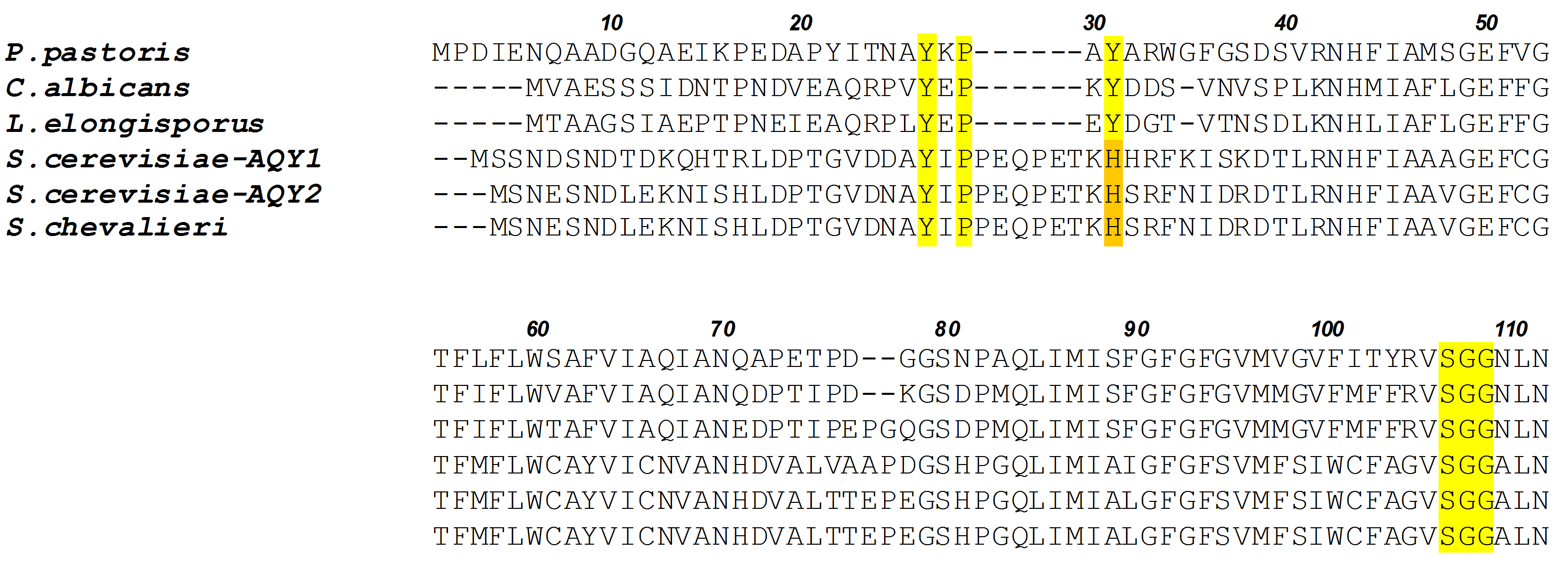

Supplement: Figure S2 — Sequence alignment of orthodox aquaporins from different yeast species. The sequence numbering corresponds to Aqy1 from P. pastoris. The residues marked yellow are potentially involved in the gating mechanism. The C-terminal parts of the sequences are not shown. The alignment was made with ClustalW. (0.24 MB TIF) [file pbio.1000130.s002.tif]

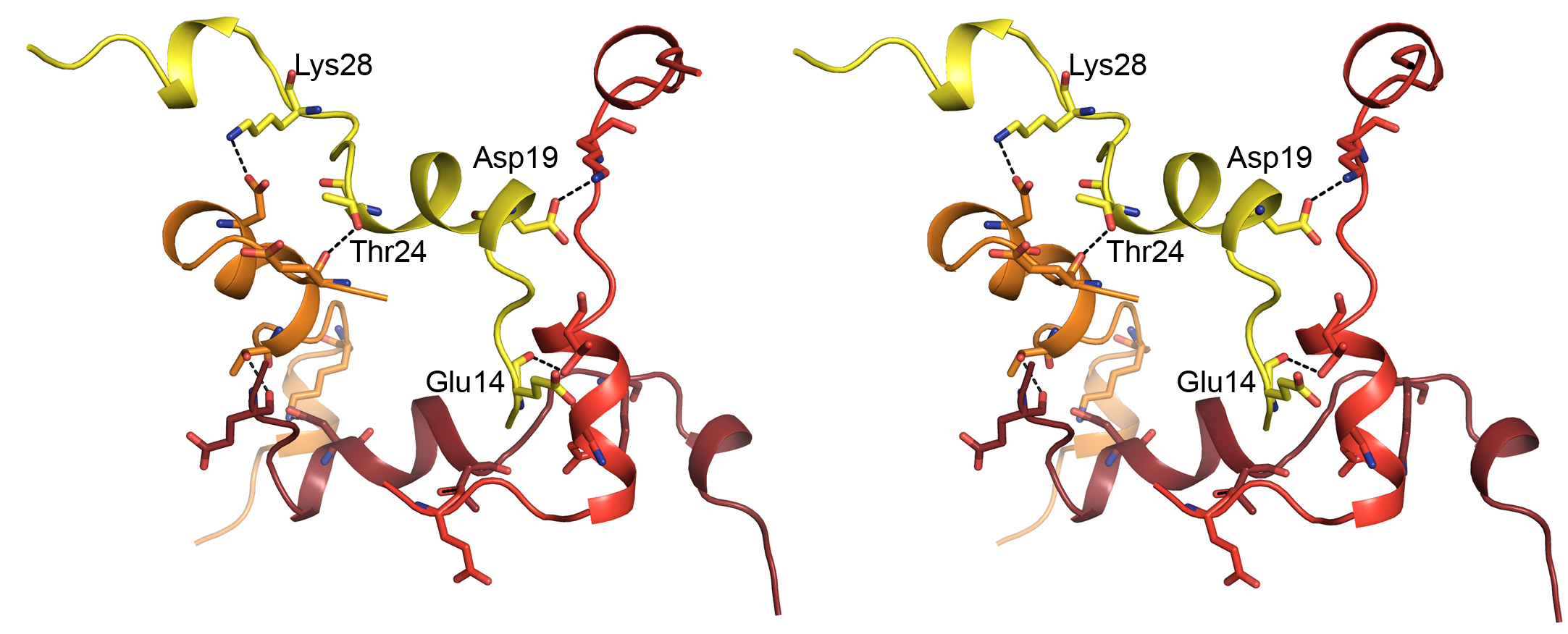

Supplement: Figure S3 — Stereo-view of the N-terminal helical bundle. Aqy1 has an N-terminal extension of 34 residues compared with the human homologue AQP1. This extension forms into two short α-helices. The N termini from the four subunits of the tetramer twist around each other and form an N-terminal bundle positioned on the cytoplasmic side of the membrane. The interactions between the N termini are shown in stereo-view. In addition to the interactions shown in this figure, the N termini also interact with loop D and the C terminus of the neighbouring subunits. (0.93 MB TIF) [file pbio.1000130.s003.tif]

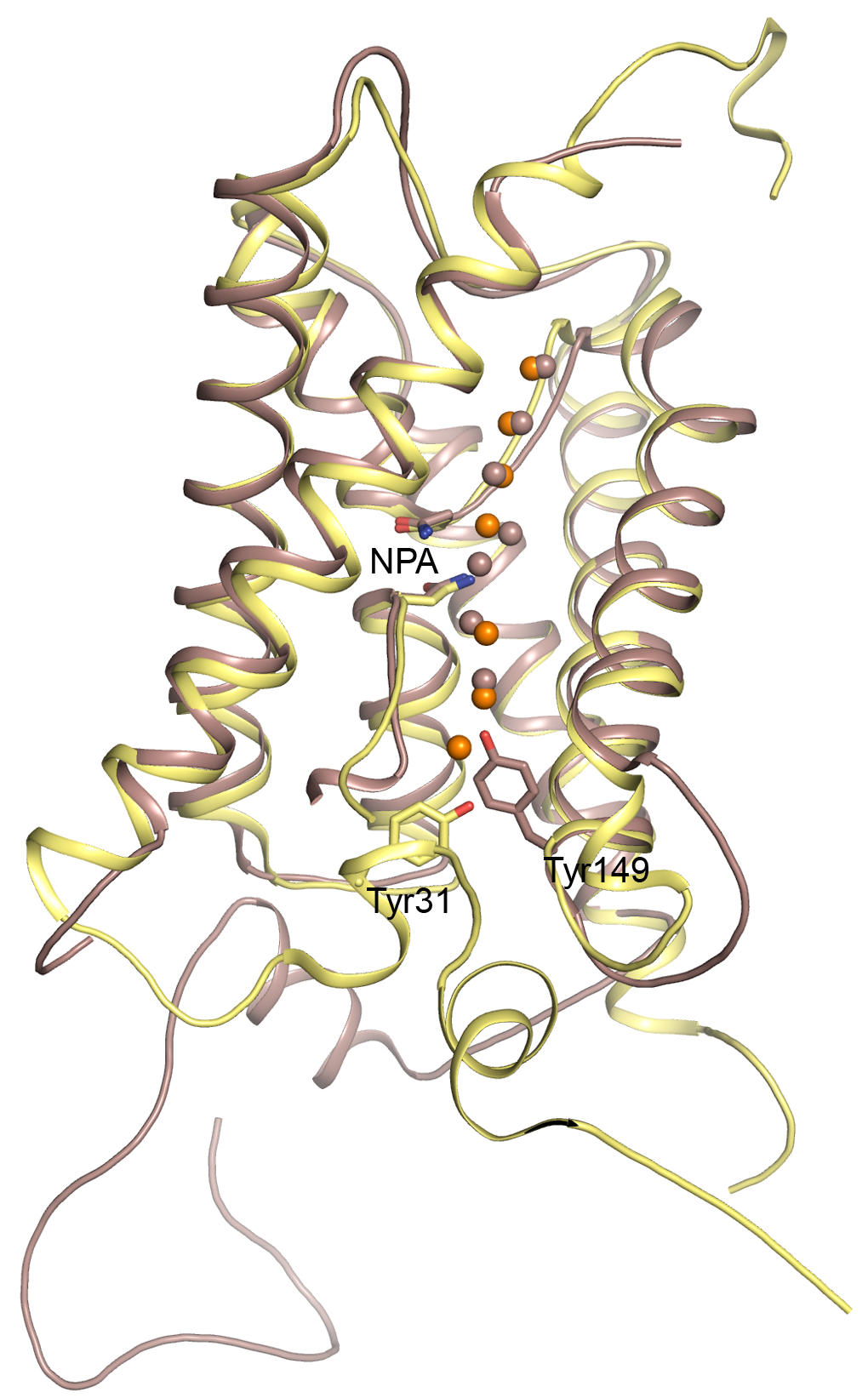

Supplement: Figure S4 — The water channels of yeast Aqy1 and mammalian AQP0 are blocked in similar fashion by a tyrosine residue. Tyr31 in Aqy1 (yellow) and Tyr149 in AQP0 (light brown, PDB code 2b6o) are shown as sticks. Water molecules found within the channel of Aqy1 are shown as orange spheres, whereas light brown spheres show water molecules found in the pore of AQP0. For clarity, only aspargine residues from the NPA regions are shown in sticks representation. (0.86 MB TIF) [file pbio.1000130.s004.tif]

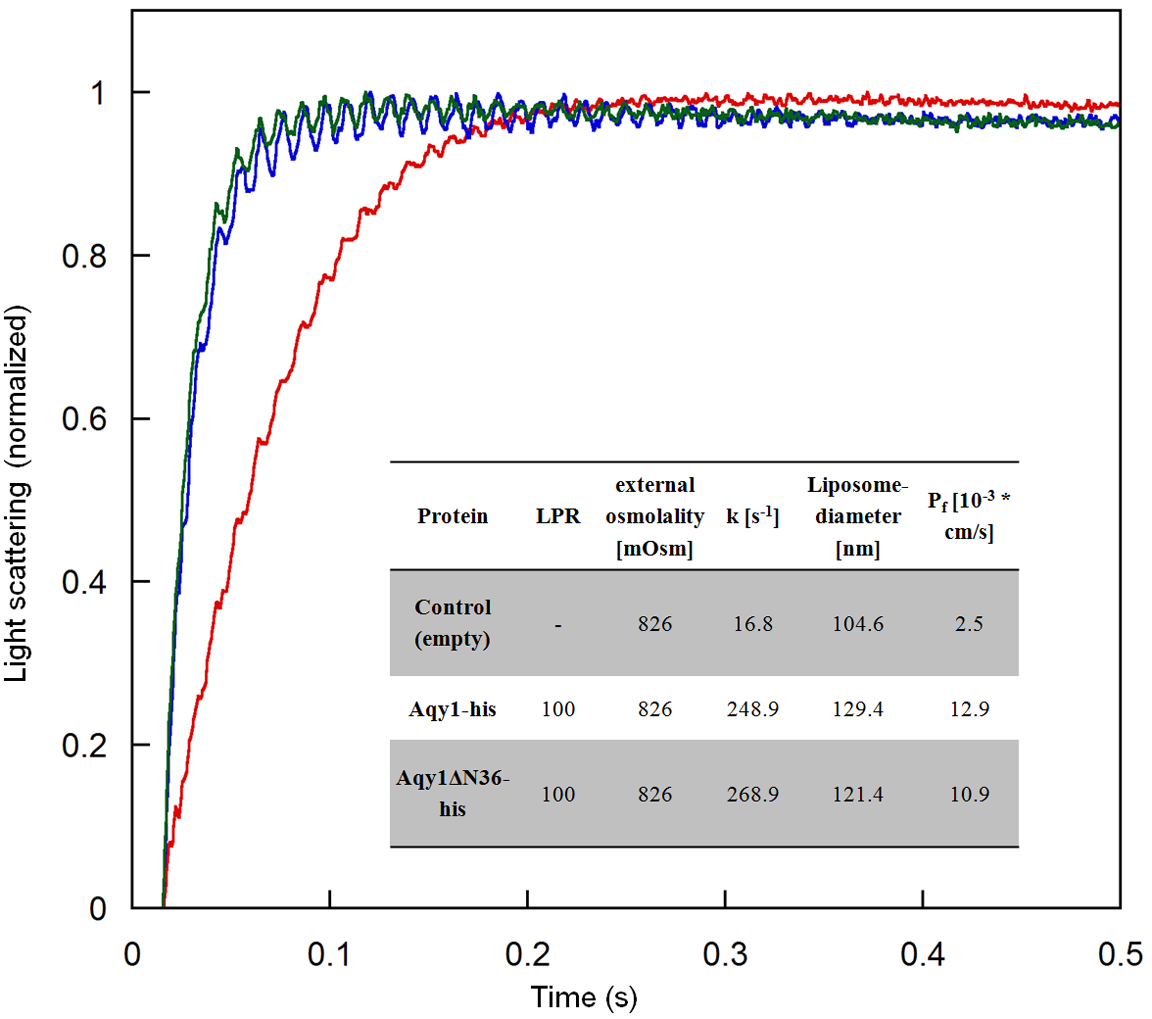

Supplement: Figure S5 — Water transport assay using light scattering spectroscopy and proteoliposomes. Change in light scattering (normalized) is plotted against time of Aqy1-his (green) and Aqy1ΔN36-his (blue) protein reconstituted into liposomes upon 1:1 mixing with an 826-mOsm external hyperosmotic sorbitol solution, compared to a control of empty liposomes (red). The inset shows the physical data obtained from curve fitting to a double exponential function. Data were derived from an average of ten traces and the rate constant values (k) of the dominant fraction were used for calculation of the permeability coefficients P f. Liposome sizes were determined by dynamic light scattering, the first cumulant value is given. Both constructs appear to be fully open when reconstituted into liposomes. In contrast, wild-type Aqy1 has a water-transport activity only one-sixth of that observed for Aqy1ΔN36 in the P. pastoris spheroplast assay (Figure 3A). The increase in activity of wild-type Aqy1 when reconstituted into proteoliposomes may be due to their high curvature triggering mechanosensitive opening of the channel (Figure 6). (0.29 MB TIF) [file pbio.1000130.s005.tif]

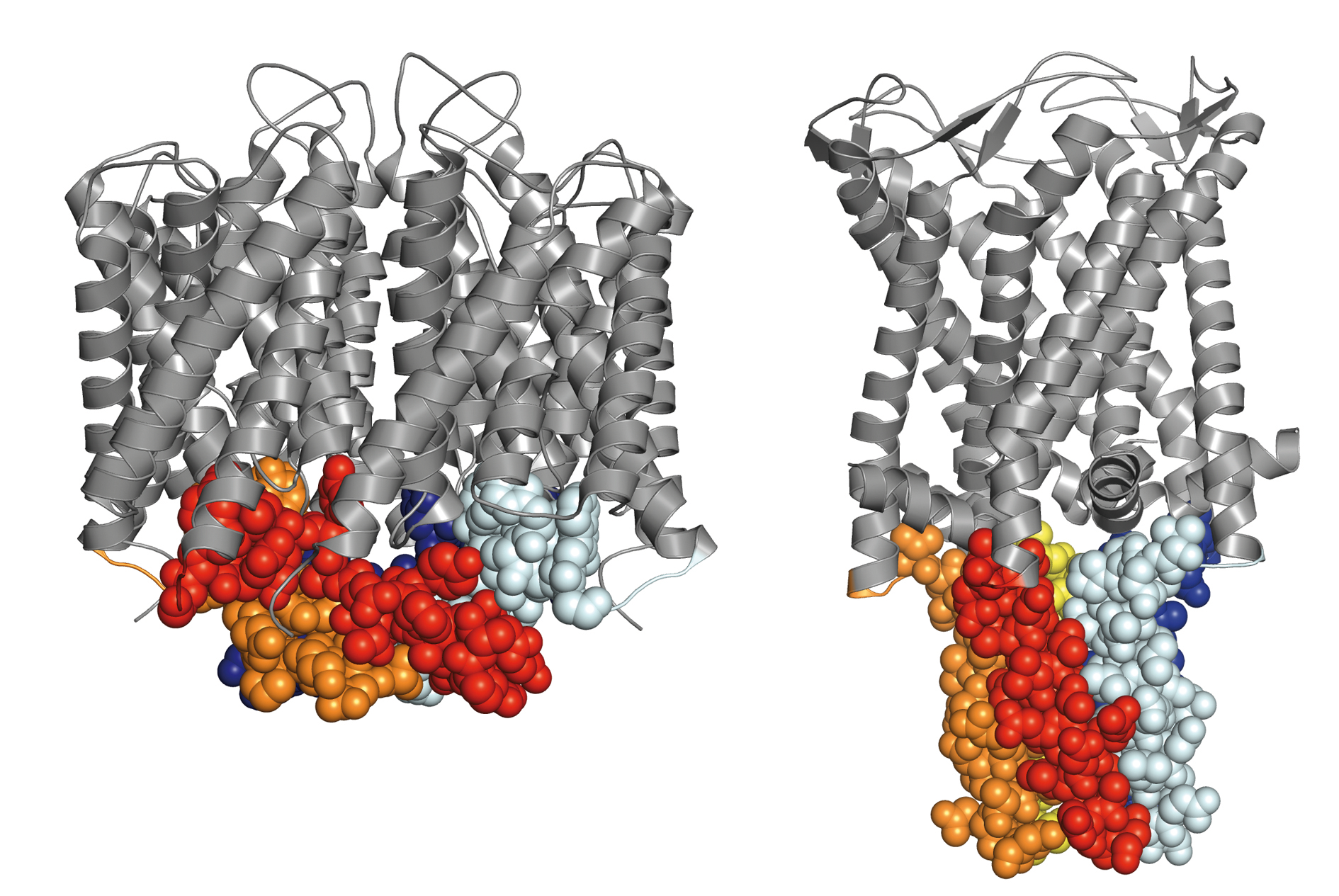

Supplement: Figure S6 — Side by side comparison of the structure of Aqy1 and MscL. The N-terminal bundle of Aqy1 (left) is reminiscent of the N-terminal bundle observed in the structure of the mechanosensitive gated ion channel MscL (right). Both structures are shown parallel with membrane. (2.20 MB TIF) [file pbio.1000130.s006.tif]

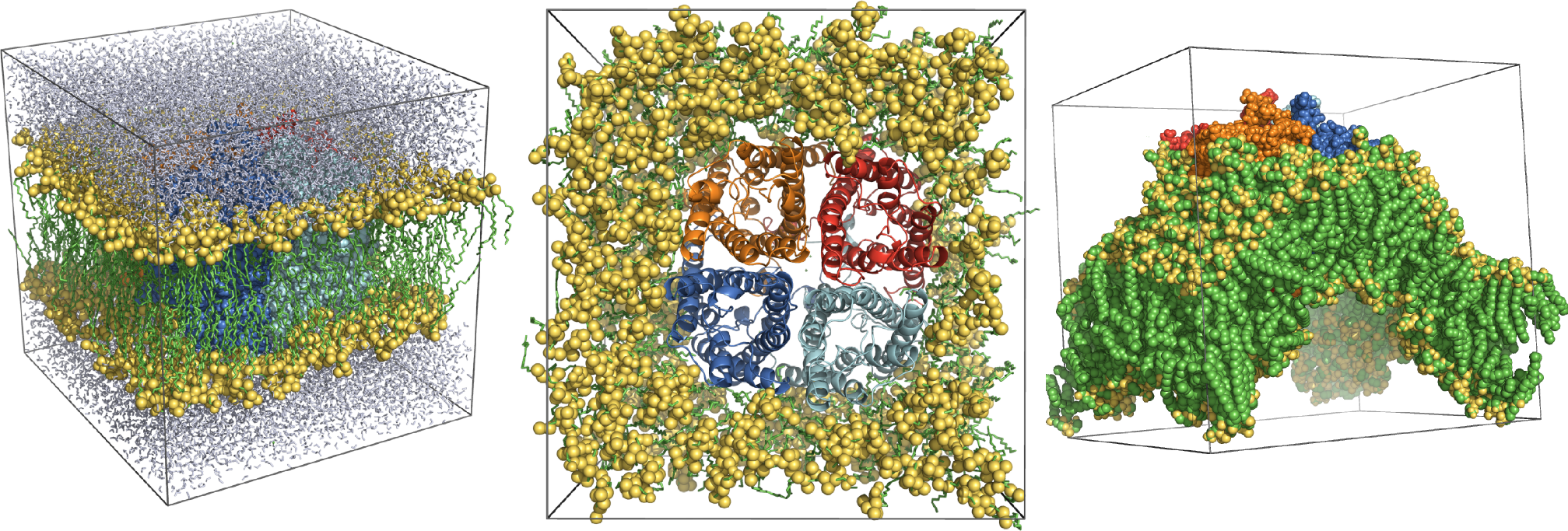

Supplement: Figure S7 — Molecular dynamics simulations of Aqy1. Simulation boxes showing the tetramer (blue, orange, red, and cyan), fully embedded in a lipid bilayer (yellow head groups and green tails) and solvated by water (blue, white), for the simulations without (left and middle panels, showing side and top views, respectively) and with (right panel) an induced bending of the membrane. (2.54 MB TIF) [file pbio.1000130.s007.tif]

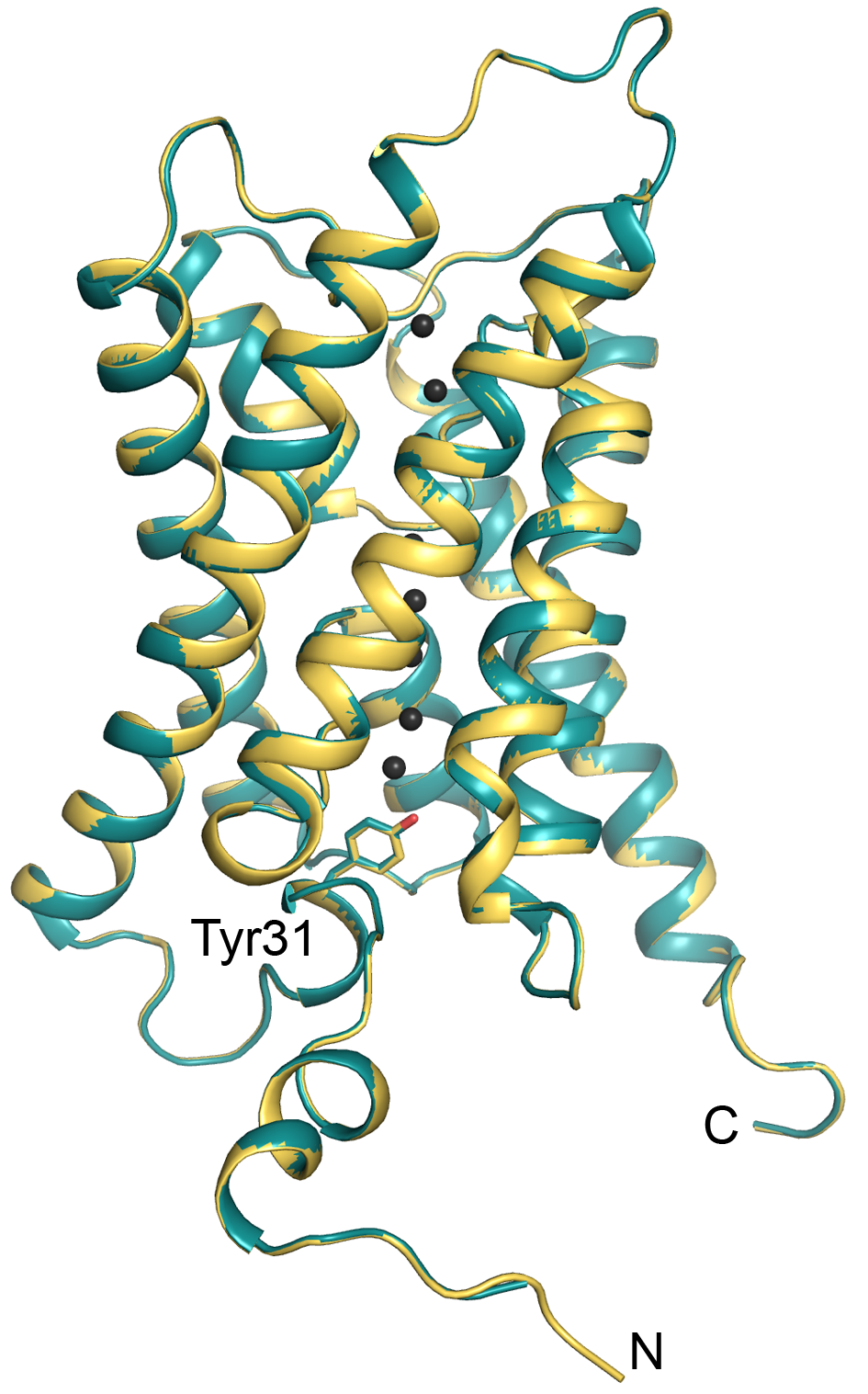

Supplement: Figure S8 — Superposition of the Aqy1 structures crystallized at pH 3.5 and pH 8.0. The crystal structures of the P. pastoris Aqy1 crystallized at pH 3.5 (yellow) and pH 8 (green) respectively show identical closed conformation. The RMS deviation is 0.08 Å for 248 Cα atoms, and 0.4 Å using 1,969 atoms of the structure. This is a strong indication that Aqy1 is not regulated by pH. (0.78 MB TIF) [file pbio.1000130.s008.tif]

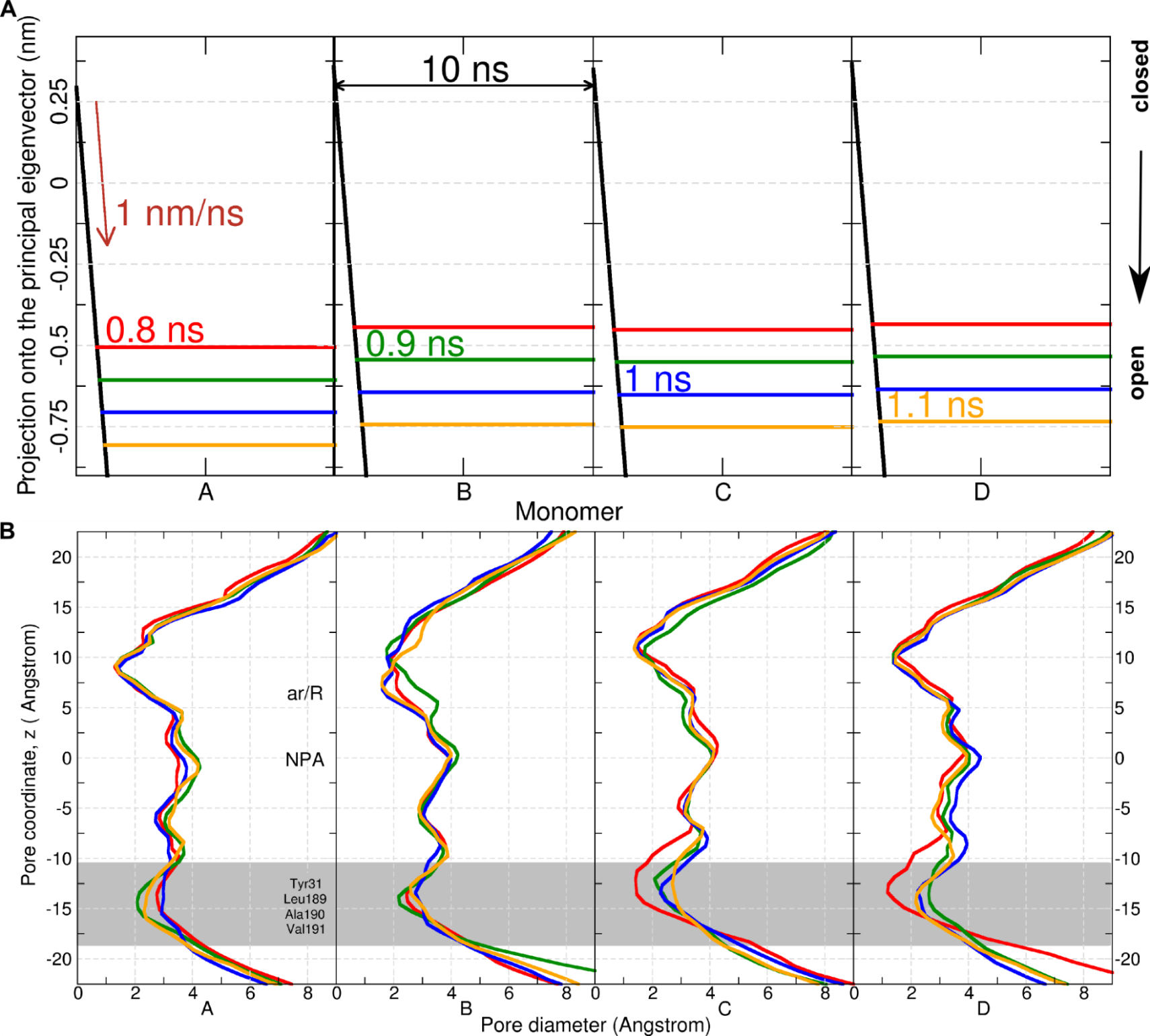

Supplement: Figure S9 — Essential dynamics simulations of Aqy1. (A) Projections of all trajectories onto the principal eigenvector found in the PCA analysis (see Figure 6C). Initially, for each monomer the driving velocity was 1 nm/ns (black lines). Then, after 0.8 ns (red), 0.9 ns (green), 1.0 ns (blue), and 1.1 ns (orange), the driving velocity was set to 0 nm/ns. (B) Averaged pore diameter profiles for each one of the four monomers, during the second part of the simulation (driving velocity equals 0 nm/ns), after 0.8 ns (red), 0.9 ns (green), 1.0 ns (blue), and 1.1 ns (orange). The pore widens near Tyr31 to values larger than 2 Å for 14 of the 16 cases, indicating that the collective coordinate represented by the first eigenvector is indeed responsible of the gating conformational change, and that the opening motions taking place at the gate of the pore are reproducible separately in the four monomers. (0.68 MB TIF) [file pbio.1000130.s009.tif]

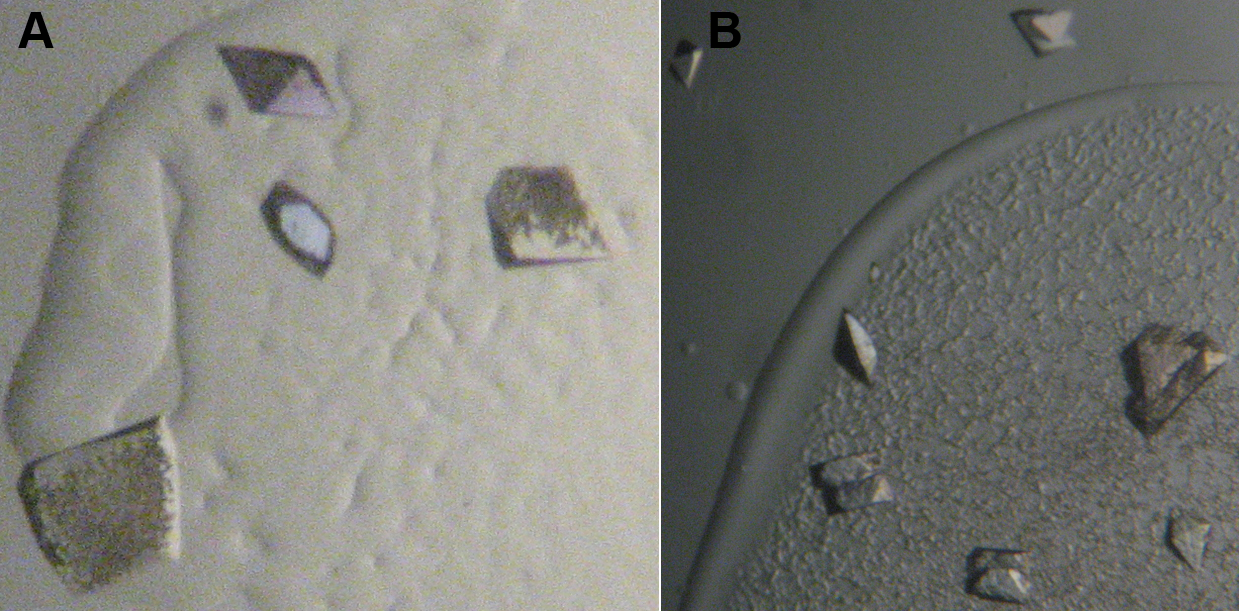

Supplement: Figure S10 — Crystals of Aqy1 from pH 3.5 and 8.0. (A) shows Crystal 1 (pH 3.5, 1.15 Å) and (B) shows Crystal2 (pH 8.0, 1.4 Å). (1.72 MB TIF) [file pbio.1000130.s010.tif]

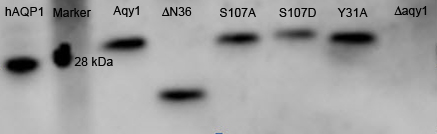

Supplement: Figure S11 — Western blot of P. pastoris membrane fractions used for the spheroplast water transport assay showing the expression levels of the respective proteins. The same amount of membrane was loaded for all constructs, except for hAQP1, for which 30× as much membrane was loaded to achieve a comparable signal. All other proteins are expressed at comparable levels. Although the S107D Aqy1 mutant may be expressed at slightly lower levels than the other Aqy1 constructs, this mutant shows a significantly increased water-transport activity in the P. pastoris spheroplast assay when compared with overproduced wild-type Aqy1. The major conclusions of the spheroplast assay (Figures 3A and 4A), that wild-type Aqy1 has a significantly lower water-transport activity than the other Aqy1 constructs, is thus unaffected by normalization against the protein yield. (0.05 MB TIF) [file pbio.1000130.s011.tif]
