# Supplementary material for: Crystal Structure of a Yeast Aquaporin at 1.15 Å Reveals a Novel Gating Mechanism
Source: PLoS Biol. 2009 Jun 16;7(6):e1000130. doi: 10.1371/journal.pbio.1000130 (PMC2688079; doi:10.1371/journal.pbio.1000130)
Supplement: Table S1 — Primers used for cloning. Nucleic acid sequences for primers used for creating the AQY1 deficient P. pastoris strain as well as strains overexpressing Aqy1 and mutants of Aqy1. (0.03 MB DOC) [file pbio.1000130.s012.doc]

| **Primer #** | **Primer sequence (5’-3’-direction)** |
| --- | --- |
| **A** | TAGACTGACTGCAGACAATCCTGACAACCAGCA |
| **B** | AGCCAGTTCTGCAGCGTTAGTGTTCGGTTTCCA |
| **C** | ATGCCTGACATTGAAAACC |
| **D** | AGCATCTGAATCTTGGCCT |
| **E** | TGCAGAATTCAAAATGCCTGACATTGAAAACC |
| **F** | TGCAGAATTCAAAATGGGTTCGGACTCT |
| **G** | GTACTCTAGATCAGTGATGGTGGTGATGGTGAGCATCTGAATCTTGGC |
