# Supplementary material for: Crystal Structure of a Yeast Aquaporin at 1.15 Å Reveals a Novel Gating Mechanism
Source: PLoS Biol. 2009 Jun 16;7(6):e1000130. doi: 10.1371/journal.pbio.1000130 (PMC2688079; doi:10.1371/journal.pbio.1000130)
Supplement: Text S1 — Supplementary information on molecular dynamics simulations. (0.07 MB DOC) [file pbio.1000130.s013.doc]

**Supplementary Methods for:**

**Crystal Structure of a Yeast Aquaporin at 1.15 Å Reveals a Novel Gating Mechanism**

Gerhard Fischer^1^*, Urszula Kosinska-Eriksson^1^*, Camilo Aponte-Santamaría^2^, Madelene Palmgren^3^, Cecilia Geijer^3^, Kristina Hedfalk^1^, Stefan Hohmann^3^, Bert L. de Groot^2^, Richard Neutze^1^, Karin Lindkvist-Petersson^3^

^1^Department of Chemistry, Biochemistry and Biophysics, University of Gothenburg, Box 462,

S-405 30 Göteborg, Sweden.

^2^Computational Biomolecular Dynamics Group, Max Planck Institute for Biophysical Chemistry, Am Fassberg 11, 37077 D-Göttingen, Germany.

^3^Department of Cell and Molecular Biology, University of Gothenburg, Box 462, S-405 30 Göteborg, Sweden.

*these authors contributed equally to this work.

Correspondence should be addressed to K.L. ([Karin.Lindkvist@gu.se](mailto:Karin.Lindkvist@gu.se)) or R.N. ([Richard.Neutze@chem.gu.se](mailto:Richard.Neutze@chem.gu.se))

**Molecular dynamics simulations*.*** Molecular dynamics simulations were carried out starting with the Aqy1 tetramer in a fully solvated palmitoyloleoylphosphatidylethanolamine (POPE) lipid bilayer, following the same approach explained in de Groot *et al.* [1]. and Hub *et al.* [2]. Five different simulations were carried out: first, control simulations under equilibrium conditions, without exerting external forces over the membrane (I); second, mutating Ser107 into Asp, mimicking a putative phosphorylated state (II); third, mutating Tyr31 into Ala (III); fourth, inducing a surface tension onto the membrane (IV), and fifth, bending the membrane towards the cytoplasmic side (V). Mutations for the simulations II and III were carried out with the WHAT IF software [3]. The simulation boxes contain the protein tetramer, 268 POPE lipids (361 for the bent membrane in simulation V) and 18580 (30842 for the bent membrane in simulation V) SPC water molecules [4]. Crystallographic water molecules were kept in the structures, and chloride ions were added to neutralize the simulation systems. The OPLS all-atom force field [5,6] was used for the protein, and lipid parameters were taken from Berger *et al*. [7]. The simulations were carried out by using the GROMACS simulation software [8,9]. Electrostatic interactions were calculated with the particle-mesh Ewald method [10,11]. Short-range repulsive and attractive interactions were described by a Lennard–Jones potential, which was cut off at 1.0 nm. The Settle algorithm [12] was used to constrain bond lengths and angles of water molecules, and Lincs [13] was used to constrain all other bond lengths, allowing a time step of 2 fs. The temperature was kept constant by weakly coupling the protein, lipids and water molecules separately to a heat bath at 300 K [14] with a coupling constant t = 0.01 ps. The pressure was kept constant by weakly coupling the system to a semiisotropic pressure bath at 1 bar with a coupling constant of t = 1 ps. To induce a surface tension in the simulation IV, the pressure in the direction parallel to the membrane surface (xy plane) was increased to 10 bar, and the pressure in the direction normal to the membrane was kept at 1 bar. To bend the membrane in the simulation V, an external force was exerted on lipids located at distances larger than 5 nm from the centre of the tetramer along the z coordinate, resulting in a net constant acceleration of 0.01 nmps^-2^. A compensation force was applied to the protein to prevent net acceleration of the simulation box. All simulations were equilibrated for 1 ns before production. During this time the coordinates of the protein were harmonically restrained, with a harmonic force constant of 1000 kJmol^-1^nm^-2^. The simulation length in the simulations I to IV was 100 ns whereas in simulation V it was 10 ns. Pore diameter profiles were obtained with the HOLE software [15], averaging over several snapshots taken each 50 ps in a time window given by t_start_ - t_end_ = 95 ns - 100 ns, 95 ns - 100 ns, 50 ns - 55 ns, 21 ns - 25 ns and 6 ns – 10 ns, for the simulations I to V, respectively. Free energy profiles were computed using the formula G(z)=-k_b_Tln<n(z)>, where k_b_ is the Boltzmann constant, T is the room temperature and <n(z)> is the average (over the whole trajectory) of the number of waters at the z position along the pore. Phe92 was observed to occasionally flip and block the pore in between the NPA and the ar/R region. These motions increased the energy barrier for water permeation in simulation II. The significance of this motion is unclear, and it was found to be independent of the opening events taking place near Tyr31. A principal component analysis, consisting of the calculation and diagonalization of the covariance matrix for all the coordinates of the backbone atoms of the lower part of helices four, five and six, and loop D, accumulated over the whole trajectory [16], was carried out using the GROMACS package tools.

To investigate the correlation between the conformational changes taking place in the gate of the pore and the collective coordinate represented by the first eigenvector (obtained in the PCA analysis), a series of 10 ns essential dynamics simulations were carried out [17]. The backbone atoms of lower parts of helices four, five and six, and the loop D were forced to move along the principal eigenvector found in the PCA analysis (see Fig. 6c), with a constant driving velocity of 1 nm/ns starting from the closed conformation. After 0.8 ns, 0.9 ns, 1.0 ns and 1.1 ns the driving velocity was set to zero nm/ns, and the atoms were constrained to maintain the projection value constant until the end of the simulation. These simulations were also carried out with the GROMACS software tools.

**References**

1. de Groot BL, Grubmuller H (2001) Water permeation across biological membranes: mechanism and dynamics of aquaporin-1 and GlpF. Science 294: 2353-2357.

2. Hub JS, de Groot BL (2008) Mechanism of selectivity in aquaporins and aquaglyceroporins. Proc Natl Acad Sci U S A 105: 1198-1203.

3. Vriend G (1990) WHAT IF: a molecular modeling and drug design program. J Mol Graph 8: 52-56, 29.

4. Berendsen HJC, Postma JPM, van der Gunsteren WF, Hermans J (1981) Intermolecular Forces; Pullman B, editor. Dordrecht, Netherlands: Reidel.

5. Kaminski GA, Friesner RA, Tirado-Rives J, Jorgensen WL (2001) Evaluation and reparametrization of the OPLS-AA force field for proteins via comparison with accurate quantum chemical calculations on peptides. Journal of Physical Chemistry B 105: 6474-6487.

6. Jorgensen WL, Maxwell DS, TiradoRives J (1996) Development and testing of the OPLS all-atom force field on conformational energetics and properties of organic liquids. Journal of the American Chemical Society 118: 11225-11236.

7. Berger O, Edholm O, Jahnig F (1997) Molecular dynamics simulations of a fluid bilayer of dipalmitoylphosphatidylcholine at full hydration, constant pressure, and constant temperature. Biophysical Journal 72: 2002-2013.

8. Van der Spoel D, Lindahl E, Hess B, Groenhof G, Mark AE, et al. (2005) GROMACS: Fast, flexible, and free. Journal of Computational Chemistry 26: 1701-1718.

9. Lindahl E, Hess B, van der Spoel D (2001) GROMACS 3.0: a package for molecular simulation and trajectory analysis. Journal of Molecular Modeling 7: 306-317.

10. Essmann U, Perera L, Berkowitz ML, Darden T, Lee H, et al. (1995) A Smooth Particle Mesh Ewald Method. Journal of Chemical Physics 103: 8577-8593.

11. Darden T, York D, Pedersen L (1993) Particle Mesh Ewald - an N.Log(N) Method for Ewald Sums in Large Systems. Journal of Chemical Physics 98: 10089-10092.

12. Miyamoto S, Kollman PA (1992) Settle - an Analytical Version of the Shake and Rattle Algorithm for Rigid Water Models. Journal of Computational Chemistry 13: 952-962.

13. Hess B, Bekker H, Berendsen HJC, Fraaije JGEM (1997) LINCS: A linear constraint solver for molecular simulations. Journal of Computational Chemistry 18: 1463-1472.

14. Berendsen HJC, Postma JPM, Van der Gunsteren WF, DiNola A, Haak JR (1984) Moldecular dynamics with coupling to an external bath. J Chem Phys 81: 3684.

15. Smart OS, Goodfellow JM, Wallace BA (1993) The pore dimensions of gramicidin A. Biophys J 65: 2455-2460.

16. Amadei A, Linssen AB, Berendsen HJ (1993) Essential dynamics of proteins. Proteins 17: 412-425.
